# Anti-inflammatory Activity and Phytochemical Profile of *Galinsoga Parviflora* Cav.

**DOI:** 10.3390/molecules23092133

**Published:** 2018-08-24

**Authors:** Elżbieta Studzińska-Sroka, Marlena Dudek-Makuch, Justyna Chanaj-Kaczmarek, Natasza Czepulis, Katarzyna Korybalska, Rafał Rutkowski, Joanna Łuczak, Karolina Grabowska, Wiesława Bylka, Janusz Witowski

**Affiliations:** 1 Department of Pharmacognosy, Poznan University of Medical Sciences, Swiecickiego 4, 60-781 Poznan, Poland; dudum@poczta.onet.pl (M.D.-M.); justynachanaj@wp.pl (J.C.-K.); wieslawabylka@tlen.pl (W.B.); 2 Department of Pathophysiology, Poznan University of Medical Sciences, Rokietnicka 8, 60-806 Poznan, Poland; czepulis@ump.edu.pl (N.C.); koryb@ump.edu.pl (K.K.); rrutkowski@ump.edu.pl (R.R.); jgrzelczak@ump.edu.pl (J.Ł.); jwitow@ump.edu.pl (J.W.); 3 Department of Pharmacognosy, Pharmaceutical Faculty, Medical College, Jagiellonian University, Medyczna 9, 30-688 Cracow, Poland; kgrabowska@cm-uj.krakow.pl

**Keywords:** gallant soldier, polyphenols, anti-inflammatory, antioxidant, anti-hyaluronidase activity, endothelium, IL-6

## Abstract

The objective of this study was to evaluate the usefulness of a hydroalcoholic extract from *Galinsoga parviflora* herb (GP) in some aspects of the endothelial cell function necessary for anti-inflammatory activity and wound healing and relate these to the GP phytochemical profile. This study demonstrated that the GP extract caused a dose-dependent reduction of IL-6 secretion on IL-1β-stimulated endothelial cells. The IL-6 release was decreased to 33% ± 9% while this did not influence the IL-6 secretion without stimulation. Additionally, the GP extract exhibited an anti-hyaluronidase activity (IC_50_ = 0.47 mg/mL), which was evidently stronger than the positive control kaempferol (IC_50_ = 0.78 mg/mL) as well as a moderate and concentration-dependent, antioxidant activity. The results of the scratch assay showed that exposure of the endothelial cells to GP induced complete healing of the damage after 12 h of the study. The phytochemical profile of the extract was studied by using spectrophotometric (total amount of polyphenols and flavonoids) and UPLC (phenolic acids) methods. The main compound in the GP extract was a chlorogenic acid (2.00 ± 0.01 mg/g by UPLC). The total content of polyphenols was 98.30 ± 0.14 mg of chlorogenic acid equivalent/g of the dry herb and content of flavonoids amounted to 6.15 ± 0.41 mg quercetin equivalent/g of the dry herb. Moreover, the presence of flavonoids in *G. parviflora* was provided after their isolation and identification by spectroscopic methods. In conclusion, it demonstrated that application of GP in the treatment of skin lesions gives possibility of wound healing based on antioxidant, anti-inflammatory, and hyaluronidase-inhibiting activities of *G. parviflora* herb extract.

## 1. Introduction

Inflammation is a key protective response to tissue injury. If uncontrolled, however, it can produce some collateral damage and contribute to further tissue destruction. The inflammatory reaction is regulated at many levels by cytokines, eicosanoids, adhesion molecules, reactive oxygen, and nitrogen species. Pharmacological attempts to curtail inflammation include cytokine inhibitors, soluble receptors or antagonists, antioxidants, and pro-resolving mediators.

Many medicinal plants used successfully in folk medicine display anti-inflammatory properties, albeit the exact molecular mechanisms by which they produce these beneficial effects are often poorly defined. It was proven that they cause transcriptional or post-transcriptional control of gene expression, inhibition of pro-inflammatory cytokines and eicosanoids, and/or antioxidant activity [1].

The gallant soldier (*Galinsoga parviflora* Cav.) (GP) is a cosmopolitan annual herb from the *Asteraceae* family. Fresh leaves and juice of GP have been used in folk medicine throughout the world to treat dermatological disorders including eczema, lichen, and non-healing and/or bleeding wounds [2]. Less often GP is administered orally to cure the common cold, the flu, and cold sores. GP has been also used as an anti-scurvy agent due to the high level of vitamin C. Moreover, GP can reduce the activation of an inflammatory response and possesses analgesic properties. The aqueous extracts of aerial parts of GP have protective effects against UV irradiation damages [2,3,4,5]. The extracts of the aerial part of GP have been found to exert significant antibacterial, antifungal [6], and antiviral activities [7]. In turn, essential oil from GP can inhibit the growth of *S. aureus* and *B. cereus* [8]. Various chemical compounds and extracts of gallant soldier have shown α-glucosidase, hepato-protective, nematicidal, and hypoglycemic activities. The use of GP as food by humans for making salad and soups in Latin and North America proves that the plant is non-toxic [2,3,4,5].

The aim of the present study was to examine anti-inflammatory, anti-hyaluronidase, and anti-antioxidant activity of hydroalcoholic extract of GP and to evaluate the healing process (in vitro) in regards to the GP phytochemical profile.

## 2. Results

### 2.1. Phytochemical Analysis and Quantification of the Major Compounds

#### 2.1.1. Isolation of Flavonoids

In the current study, seven known flavonoids were isolated from the ethyl acetate and water extracts of the GP herb by using cellulose column chromatography. Their structures were identified based on spectroscopic analyses (^1^H-NMR, ^13^C-NMR, and/or ESI-MS) in comparison with the literature [9,10] (see Supplementary Materials).

#### 2.1.2. UPLC Analysis of GP Extract

The identification and the quantification of phenolic acids were carried out by means of an ultra-high performance liquid chromatography supported by a photodiode array detector. The chromatograms were evaluated for linearity, limits of quantification, and recovery of each of the phenolic acids under study (Table 1).

**Figure 1:**
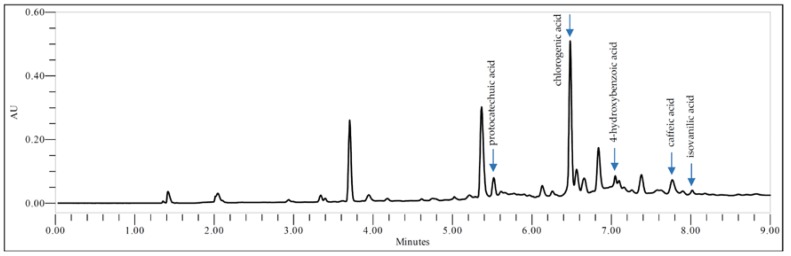
SPE-UPLC fingerprint of the *G. parviflora* extract (GP).

The UPLC analysis of the hydroalcoholic GP extracts demonstrated the presence of protocatechuic, chlorogenic, 4-hydroxybenzoic, caffeic, and isovanilic acids (Figure 1). The highest content was determined for chlorogenic acid (2.00 ± 0.01 mg/g of the dry herb). The other phenolic acids occurred in the lower amounts (µg/g of the dry herb): protocatechuic acid (200.32 ± 4.3), 4-hydroxybenzoic acid (100.43 ± 0.2), caffeic acid (120.54 ± 2.8), and isovanilic acid (60.38 ± 1.6).

#### 2.1.3. Total Phenolic and Flavonoid Content

Total phenolic content (TPC) and total flavonoid (TFC) content of the GP extract were 98.30 ± 0.14 mg chlorogenic acid equivalent/g of the dry herb and 6.15 ± 0.41 mg/quercetin equivalent/g of the dry herb, respectively.

### 2.2. Bioactivity Assay

#### 2.2.1. LAL Assay

A concentration of endotoxins of <0.01 ng/mL in GP extract was considered acceptable and this extract was used for further in vitro studies. The concentration of GP was <1.0 mg/mL.

#### 2.2.2. Viability and Proliferation Assay

**Figure 2:**
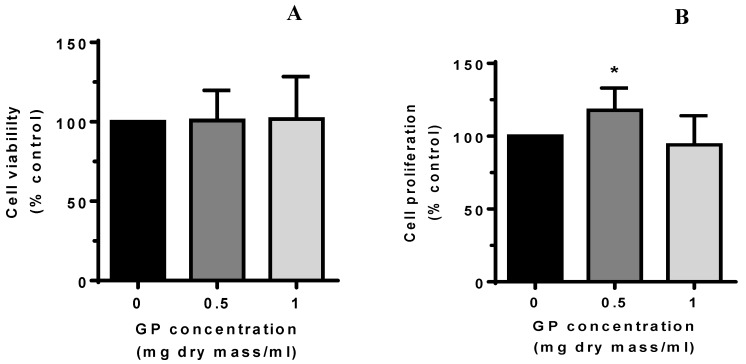
Effect of the *G. parviflora* extract (GP) on endothelial cell viability (**A**) and proliferation (**B**). Cells were treated with the GP extract or vehicle control for 24 h and was then assessed for viability (Trypan blue exclusion test) and proliferation (MTT test). The data were derived from three independent experiments. The data expressed as mean ± SD. Asterisks represent a significant difference compared to control cells (* *p* < 0.05).

Exposure of endothelial cells to GP extract at concentrations up to 1 mg/mL did not impair cell viability, which is measured by the trypan blue exclusion test (Figure 2A). The GP extract at a dose of 0.5 mg/mL also slightly stimulated endothelial cell proliferation. However, the growth of cells treated with the GP concentration at twice as high an amount did not have any significant effect (Figure 2B).

#### 2.2.3. Cytokine Measurements

**Figure 3:**
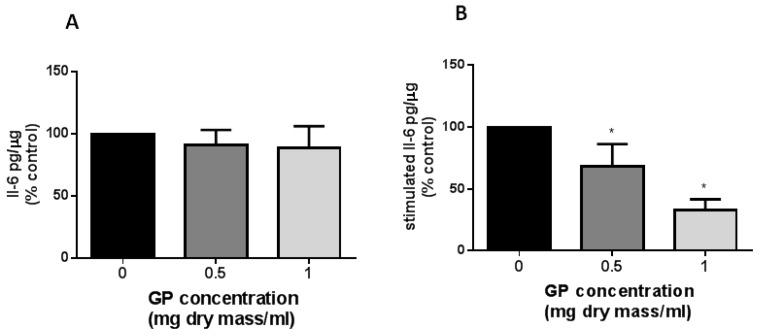
Effect of *G. parviflora* extract (GP) on IL-6 release by endothelial cells. Cells were treated with a GP extract in the absence (**A**) or presence (**B**) of Il-1β (1 ng/mL) for 24 h. The data were derived from four independent experiments and are expressed as a parentage of control (mean ± SD). The IL-6 release was detected as pg/µg cell protein. Asterisks represent a significant difference when compared to control cells (* *p* < 0.5).

None of the tested concentrations of the GP extract changed constitutive IL-6 release by endothelial cells (Figure 3A). In contrast, the exposure to the GP resulted in a dose-dependent decrease in IL-1β-stimulated IL-6 secretion (Figure 3B). The highest concentration of the GP caused the release of Il-1β-stimulated IL-6, which was reduced to 33% ± 9%.

#### 2.2.4. Anti-Hyaluronidase Activity

**Figure 4:**
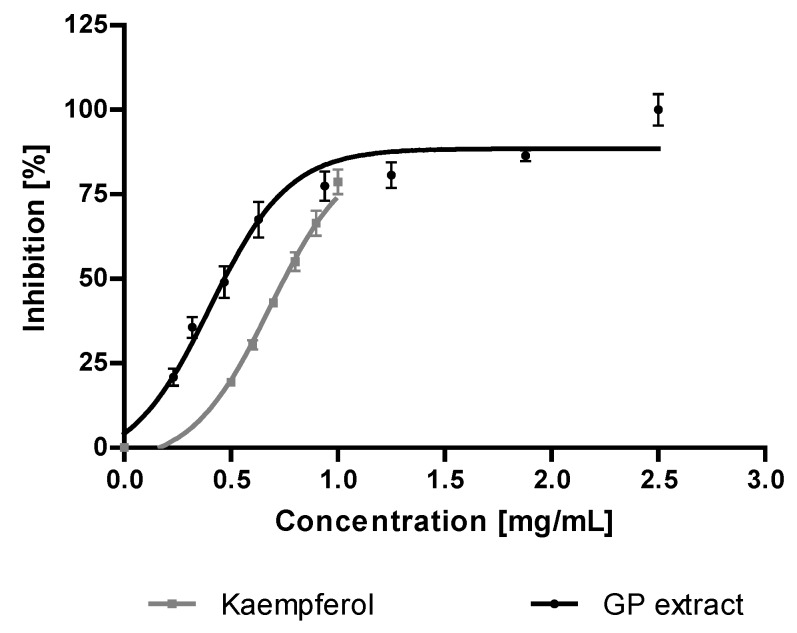
Anti-hyaluronidase activity of *G. parviflora* extract (GP) and the reference substance. Results are presented as mean values ± SD (*n* = 3 × 2) represented by error bars, IC_50_, 50% inhibition of enzyme activity.

The results of an anti-hyaluronidase activity assay are shown in Figure 4. The study showed that the GP extract (IC_50_ = 0.47 mg/mL) exhibited a stronger activity than the positive control kaempferol (IC_50_ = 0.78 mg/mL).

#### 2.2.5. Antioxidant Activity

The FRAP assay revealed a potent antioxidant property of the GP extract with estimated IC_0.5_ = 498.2 µg/mL of the dry herb when compared to IC_0.5_ = 44.40 µg/mL for l-ascorbic acid.

#### 2.2.6. Measurement of Reactive Oxygen Species (ROS)

**Figure 5:**
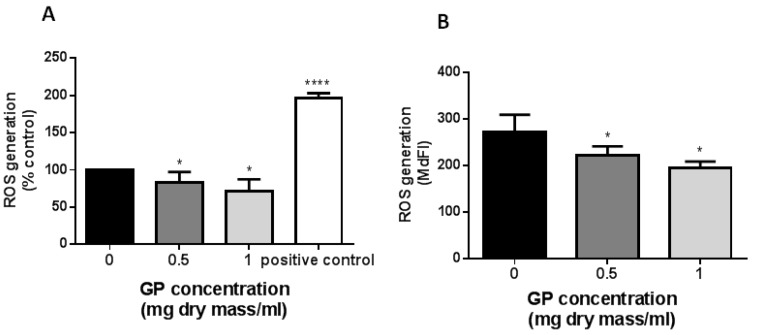
Effect of *G. parviflora* extract (GP) on endothelial ROS production. HUVECs EA.hy926 line were treated with GP extract or vehicle control for 24 h. (**A**) ROS generation were measured after H2DCFDA labeling. The data were derived from three independent experiments and are expressed as a percentage of control. The positive control is hydrogen peroxide (100 µM). Asterisks represent a significant difference compared to the control cells. (**B**) ROS generation was measured by flow cytometry after labeling with DHR. The data were derived from five independent experiments and are expressed as mean ± SD. Asterisks represent a significant difference when compared to control cells (* *p* < 0.5, **** *p* < 0.0001).

The GP extract reduced dose-dependently ROS generation by endothelial cells. In cells treated with the GP extract at 1.0 mg/mL, the level of intracellular ROS was decreased by approximately 30%. The effect of the GP extract was clearly seen in cells labeled independently with two different dyes H_2_DCFDA and DHR using an in vitro culture (Figure 5A,B). Doing experiments with DCFDA labeling in vitro, we use as the positive control H_2_O_2_. The ROS production in response to hydrogen peroxide was 196% ± 7% when compared with control cells. The reaction to H_2_O_2_ is usually between 80% to 150% higher than that observed in the control cell HUVEC line EA.hy926 (personal observation).

#### 2.2.7. Wound Healing

**Figure 6:**
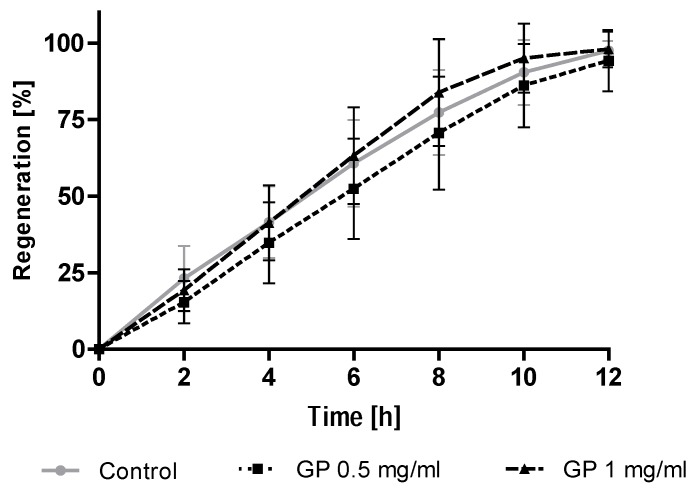
Effect of the *G. parviflora* extract (GP) on the regeneration of wound healing. Cells were treated with the GP extract or the vehicle control for 12 h and then assessed regeneration (time-lapse microscopy). The data were derived from eight independent experiments testing cell regeneration. The data expressed as a mean ± SD.

Exposure of the endothelial cells to the GP extract in concentration 0.5 mg/mL and 1 mg/mL, which caused complete healing of the damage after 12 h of the study (Figure 6). Regeneration kinetics did not differ significantly between the tested groups.

## 3. Discussion

Natural substances are often used for the treatment of skin inflammation and wound healing. One of the plants used for this purpose in folk medicine is *G. parviflora.* In our study, we verified whether the use of hydroalcoholic extract from this plant species has a scientific justification.

Defining the chemical profiles of plant extracts can justify their biological activity. Phytochemical studies of *G. parviflora* confirmed the presence of several phenolic compounds with potential biological activity.

From aerial parts of *G. parviflora* used to prepare the hydroalcoholic extract for the bioactivity assay, we isolated seven flavonoids and identified them by ESI-MS, ^1^H, and ^13^C-NMR as miquelianin, isoquercitrin, rutin, astragalin, quercimeritrin, quercetagetrin, and patulitrin. As a result of the previous LC-MS analysis of the GP extract, the different flavones, flavonols, and flavanones were also detected [2,11,12,13,14].

Our UPLC study of the hydroalcoholic GP extract revealed the presence of five phenolic acids. Apart from the protocatechuic, chlorogenic, 4-hydroxybenzoic, and caffeic, which were found in the previous studies [15,16,17]. We additionally detected the presence of isovanilic but not caffeoylglucaric acids [17]. Our results showed that chlorogenic acid is the major constituent from the group of phenolic acids in the GP extract and its total concentration was 2.00 ± 0.01 mg/g of the dry herb. The content of protocatechuic, 4-hydroxybenzoic, caffeic, and isovanilic acids amounts to (µg/g of the dry herb) 200.32 ± 4.3, 100.43 ± 0.2, 120.54 ± 2.8, and 60.38 ± 1.6, respectively. The chlorogenic acid content detected in our GP extract was comparable with the one previously determined by Ranilla et al. [16].

The analysis of the content of total polyphenols showed their high quantity in our hydroalcoholic extract. The similar analysis of methanolic and aqueous extracts from *G. parviflora*, which contained 15.3 and 24.1 mg gallic acid/g of dry herb, respectively, which was performed by Akula et al. [18]. Despite the Akula studies, the content was expressed as equivalent of gallic acid. We can affirm that the hydroalcoholic extract is a preparation rich in polyphenolic compounds. The obtained results of total flavonoids indicate their moderate content in the examined GP extract. Our results of the phytochemical analysis the GP extract provide more detailed information about flavonoid and phenolic acid profiles of this plant species.

The next step of our study was the evaluation of the biological properties of GP extract, which are important in supporting the treatment of inflammation. Inflammation is a multifactorial and multistage process that is accompanied by various factors such as the high level of interleukin production and increased hyaluronidase activity [19]. It is obvious that excessive hyaluronidase activity causes vasodilatation, boosts permeability, and, as a consequence, local redness and edema.

In the present study, we showed for the first time that the GP extract caused a dose-dependent reduction of IL-6 secretion on IL-1β-stimulated endothelial cells and the release was significantly decreased in both of the studied concentrations (Figure 3). The previous study showed that ethanolic GP extracts inhibit the arachidonic acid inflammatory pathway by blocking the activity of cyclooxygenase (COX-1) [20] and lipoxygenase (5-LOX) [18]. Moreover, GP can reduce the activation of the inflammatory response [21]. Several in vitro and in vivo models have clearly demonstrated anti-inflammatory activity of several phenolics such as chlorogenic acid, rutin, and quercetin 3-*O*-β-glucuronide, which are also present in the GP extract examined in our study [22,23,24]. The activity of these compounds resulted in decreasing the secretion of pro-inflammatory cytokines IL-6 in cells stimulated with TNF-α, LPS, and IL-1β. Therefore, it can be assumed that these GP ingredients are responsible for the anti-inflammatory effect [25,26].

Our study revealed that the GP extract completely inhibited the hyaluronidase activity at the concentration of 2.50 mg/mL (Figure 4). It has been shown that the hyaluronidase inhibitory activity is characteristic for quercetin derivatives (rutin and quercetin 3-*O*-β-glucuronide) and it is higher than that of the phenolic acids (chlorogenic and protocatechuic) [27,28,29]. The presence of rutin and the high content of phenolic compounds especially chlorogenic acid in hydroalcoholic GP extract can justify the anti-hyaluronidase activity.

Inflammation is also associated with the generation of large amounts of free radicals. Oxidative stress is capable of activating transcription factors (e.g., NF-κB) that promote the synthesis of pro-inflammatory cytokines (e.g., IL-6). In this respect, it has been demonstrated that antioxidants can reduce IL-6 and TNF-α by macrophages [30] and inhibit cyclooxygenase-2 and inducible nitric oxide synthase expression [31] as well as enhance anti-inflammatory IL-10 secretion [30]. In order to evaluate the antioxidant activity of polyphenol-rich GP extract, two used in in vitro models were applied. In our study the GP extract exhibited a moderate concentration-dependent antioxidant activity. The activity analyzed by using a FRAP assay was 11 times lower in comparison with vitamin C. The antioxidant properties of the GP extract were also confirmed by measuring ROS production by endothelial cell using DHR and DCFDA. The data demonstrated a wide spectrum of activity of the examined extract on ROS concentration in cells.

An interesting antioxidant activity of different GP extracts was also documented in other authors’ research. The study by Bazylko et al. [17] involved the scavenging of two radicals (O_2_^•−^ and H_2_O_2_) generated by fibroblasts in cell-free systems and in human skin after UV irradiation by methanolic and aqueous GP extracts. Previous studies have also indicated that GP inhibits linoleic acid oxidation and is capable of scavenging ROS [11,16,18]. Chanaj-Kaczmarek and coworkers detected a potent antioxidant activity with value IC_50_ = 47.21 µg/mL and IC_50_ = 66.38 µg/mL for decoction and 70% methanolic extract, respectively, using the ABTS assay. Additionally, the antioxidant activity was tested using a method of the *Saccharomyces cerevisiae* yeast strain without gene sod1. The decoction and 70% extract improved the viability of *S. cerevisiae* due to antioxidative activity of phenolic compounds especially phenolic acids [32]. The antioxidant properties of GP proven in our analysis as well as in the other research studies suggest that this plant species may exert a beneficial effect in the symptoms associated with inflammation.

Many reports have demonstrated that flavonoids (e.g., kaempferol, quercetin) support the acceleration of wound closure through a complex mechanism that involves inducing intercellular calcium-dependent pathways, stimulating collagen deposition, and suppressing cyclooxygenase-2 (COX-2) expression [33,34]. In the case of chlorogenic acid, a significant acceleration of keratinocyte wound closure as measured by a scratch assay has been demonstrated [35]. Moreover, a study conducted by Schmidt showed that hexane and ethanol extracts of GP promote migration and proliferation of fibroblasts and thus facilitate wound healing [21]. The results of these studies have not been confirmed in our research.

Our study, which was carried out on endothelial cells, showed that the hydroalcoholic GP extract slightly enhanced the proliferation process. Despite the high content of chlorogenic acid and the presence of flavonoids in GP extract, the improvement of wound closure was observed only after eight and 10 hours. However, the results in the scratch assay were not statistically significant.

## 4. Material and Methods

### 4.1. Chemicals and Instruments

Aluminium trichloride, formic acid, hydrochloric acid, sodium carbonate, sodium hydroxide, DMSO, and solvents used for extraction and separation flavonoids were purchased from Avantor Performance Materials Poland S.A. (Gliwice, Poland). The Folin-Ciocalteu′s phenol reagent was from Merck (Darmstadt, Germany), HPLC grade water, HPLC grade acetonitrile, acetate buffer were from the JT Baker–Avantor Performance Materials B.V. (Deventer, The Netherlands), and chlorogenic acid and kaempferol from Roth GmbH (Karlsruhe, Germany). The lysis buffer was from Promega, (Madison, WI, USA). The Protein Assay Dye Reagent was purchased from Bio-Rad (Hercules, CA, USA). The Limulus amebocyte lysate (LAL) assay was from Thermo Fisher Scientific (Rockford, IL, USA). All other chemicals were from the Sigma–Aldrich Chemical Co. (Taufkirchen, Germany). The cell culture plastics were from Nunc (Roskide, Denmark).

CF-11 cellulose (Whatman Ltd., Maidstone, Kent, UK) and Sephadex LH-20 (25–100 µ) (Sigma–Aldrich Chemical Co.) were used for column chromatography (CC). Preparative paper chromatography (PPC) (Whatman paper No. 3 MM, Whatman Ltd. Maidstone, Kent, UK) was used to separated fractions obtained after CC. Thin-layer chromatography (TLC) was performed on cellulose precoated plates (TLC Cellulose 25 Aluminium sheets 20 × 20, Merck, Darmstadt, Germany). The chromatograms were visualized under UV light at 366 nm before and after spraying with 0.1% methanolic Naturstoffreagenz A (Roth -Carl Roth GmbH + Co., Karlsruhe, Germany).

^1^H-NMR and ^13^C-NMR spectra were measured on Varian 300 MHz (compounds **2**–**4**), Bruker Avance II 400 MHz (compounds **3**,**5**) and Bruker Avance III 500 MHz (compounds **6**,**7**). Chemical shifts are given in ppm with tetramethylsilane (TMS) as an internal standard using DMSO-*d*_6_ or CD_3_-OD as solvents. The ESI-MS spectra were obtained using a Waters/Micromass (Manchester, UK) ZQ Mass spectrometer connected with HPLC (Waters typ 2690; Milford, CT, USA) and spectrometer UV Photodiode Array Detector Waters 996 (λ = 200–500 nm). ESI-MS analyses for compounds **1**–**7** were performed in positive and negative ionization models.

### 4.2. Plant Material

GP was collected (June, 2016) from the garden of the Department of Medicinal and Cosmetic Natural Products, Poznan University of Medical Sciences and dried at room temperature. The voucher specimen (No Gp-2016) was deposited at the Department of Pharmacognosy, Poznan University of Medical Sciences.

### 4.3. Extracts Preparation

The herb of GP (5.0 g) was extracted with 50% methanol (two times at 250 mL) for 20 min at 95 °C on a water bath. The extract was concentrated under a vacuum at 40 °C until it dries. The residue obtained was diluted with 50 mL of water to yield a stock solution (0.1 g dry herb/mL) that was used in further experiments. Final concentrations of the GP extract used are expressed as mg of dry herb per mL.

An extract for flavonoid isolation (from 1000 g) was prepared in the same way.

### 4.4. Phytochemical Analysis and Quantification of the Major Compounds

#### 4.4.1. Isolation of the Flavonoids

The obtained dry extract was treated with hot distilled water. Water extract was extracted successively with CHCl_3_ and then with Et_2_O and EtOAc. The ethyl acetate and water extracts were fractionated on a cellulose column (CC) with EtOAc-MeOH-H_2_O (100:6:20, *v*/*v*/*v*) as an eluent to obtain four (I, II, III, IV) and two fractions (V, VI) respectively. The fractions were collected and combined by thin layer chromatography (TLC) examination using HOAC-H_2_O (15:85, *v*/*v*) and *n*-BuOH-HOAc-H_2_O (6:1:2, *v*/*v*/*v*) as developing solvents. Compound **1** (13 mg) was isolated from fraction IV by preparative paper chromatography (PPC) using *n*-BuOH-HOAc-H_2_O (6:1:2, *v*/*v*/*v*). Fraction II and III were separated into compounds **2** (8 mg) and **3** (7 mg) by PPC using HOAC-H_2_O (30:70, *v*/*v*). Compounds **4** (8 mg) and **5** (7 mg) were isolated from fraction V by applying a cellulose column eluted by H_2_O. From the I fraction, compound **6** (13 mg) and **7** (9 mg) were isolated by using twice PPC and HOAC-H_2_O (15:85, *v*/*v*) as an eluent. All the individual compounds were re-chromatographed by CC on Sephadex LH-20 using MeOH-CHCl_3_ mixture (1:9, *v*/*v*) for elution and identified using spectroscopic analysis. Their structures were identified based on spectroscopic analyses (^1^H-NMR, ^13^C-NMR, and/or ESI-MS) in comparison with the literature [9,10] (see Supplementary Materials).

Compound **1** (7-*O*-β-glucoside of quercetagetin = quercetagetrin): ESI-MS negative: *m*/*z* 479 [M − H]^−^, *m*/*z* 317 [A − H]^−^; ESI-MS positive: *m*/*z* 481 [M + H]^+^, *m*/*z* 319 [A + H]^+^.

Compound **2** (quercetin 7-*O*-β-glucoside = quercimeritrin): ESI-MS negative: *m*/*z* 463 [M − H]^−^, *m*/*z* 301 [A − H]^−^; ESI-MS positive: *m*/*z* 465 [M + H]^+^, *m*/*z* 303 [A + H]^+^.^1^H-NMR (DMSO-*d*_6_) δ (ppm): 6.41 (d, *J* = 2.0 Hz, H-6), 6.76 (d, *J* = 2.0 Hz, H-8), 7.72 (d, *J* = 2.0 Hz, H-2′), 6.89 (d, *J* = 8.5 Hz, H-5′), 7.55 (dd, *J* = 2.0 and *J* = 8.6 Hz, H-6′), 5.07 (d, *J* = 7.2 Hz, H-1′′ glucose), ^13^C-NMR (DMSO-*d*_6_) δ (ppm): 147.90 (C-2), 136.09 (C-3), 176.01 (C-4), 160.34 (C-5), 98.73 (C-6), 162.66 (C-7), 94.23 (C-8), 155.71 (C-9), 104.64 (C-10), 121.80 (C-1′), 115.56 (C-2′), 145.05 (C-3′), 147.56 (C-4′), 115.34 (C-5′), 120.02 (C-6′), 99.85 (C-1′′), 73.10 (C-2′′), 77.13 (C-3′′), 69.53 (C-4′′), 76.38 (C-5′′), 60.59 (C-6′′).

Compound **3** (patuletin-7-*O*-β-glucopyranoside = patulitrin): ESI-MS negative: *m*/*z* 493 [M − H]^−^, *m*/*z* 331 [A − H]^−^, ESI-MS positive: *m*/*z* 495 [M + H]^+^, *m*/*z* 333 [A + H]^+^.^1^H-NMR (CD_3_-OD) δ (ppm): 6.88 (s, H-8), 7.76 (d, *J* = 1.6 Hz, H-2′), 6.88 (d, *J* = 8.7 Hz, H-5′), 7.67 (dd, *J* = 1.8 and *J* = 8.2 Hz, H-6′), 3.92 (s, OCH_3_), 5.10 (d, *J* = 7.3 Hz, H-1′′ glucose), ^13^C-NMR (DMSO-*d*_6_) δ (ppm): 147.69 (C-2), 135.80 (C-3), 176.13 (C-4), 151.04 (C-5), 131.80 (C-6), 156.36 (C-7), 93.84 (C-8), 151.39 (C-9), 104.96 (C-10), 121.84 (C-1′), 115.41 (C-2′), 145.06 (C-3′), 147.91 (C-4′), 115.58 (C-5′), 120.03 (C-6′), 60.32 (OCH_3_), 100.13 (C-1′′), 73.20 (C-2′′), 76.68 (C-3′′), 69.56 (C-4′′), 77.22 (C-5′′), 60.60 (C-6′′).

Compound **4** (quercetin-3-*O*-β-glucopyranoside = isoquercitrin): ESI-MS negative: *m*/*z* 463 [M − H]^−^, *m*/*z* 301 [A − H]^−^, ESI-MS positive: *m*/*z* 465 [M + H]^+^, *m*/*z* 303 [A + H]^+^.^1^H-NMR (DMSO-*d*_6_) δ (ppm): 12.64 (s, 5-OH), 6.19 (d, *J* = 1.9 Hz, H-6), 6.40 (d, *J* = 1.9 Hz, H-8), 7.57 (d, *J* = 2.0 Hz, H-2′), 6.84 (d, *J* = 9.0 Hz, H-5′), 7.57 (dd, *J* = 2.2 and *J* = 8.9 Hz, H-6′), 5.46 (d, *J* = 7.5 Hz, H-1′′ glucose), ^13^C-NMR (DMSO-*d*_6_) δ (ppm): 156.12 (C-2), 133.29 (C-3), 177.40 (C-4), 161.21 (C-5), 98.68 (C-6), 164.26 (C-7), 93.51 (C-8), 156.31 (C-9), 103.90 (C-10), 121.12 (C-1′), 115.20 (C-2′), 144.81 (C-3′), 148.47 (C-4′), 116.16 (C-5′), 121.58 (C-6′), 100.85 (C-1′′), 74.08 (C-2′′), 76.49(C-3′′), 69.91 (C-4′′), 77.55 (C-5′′), 60.94 (C-6′′).

Compound **5** (quercetin-3-*O*-β-glucuronide = miquelianin): ESI-MS negative: *m*/*z* 477 [M − H]^−^, *m*/*z* 301 [A − H]^−^; ESI-MS positive: *m*/*z* 479 [M + H]^+^, *m*/*z* 303 [A + H]^+^.^1^H-NMR (DMSO-*d*_6_) δ (ppm): 12.26 (s, 5-OH), 6.17 (d, *J* = 1.9 Hz, H-6), 6.36 (d, *J* = 1.9 Hz, H-8), 8.28 (d, *J* = 2.2 Hz, H-2′), 6.82 (d, *J* = 8.4 Hz, H-5′), 7.34 (dd, *J* = 2.0 and *J* = 8.4 Hz, H-6′), 5.20 (d, *J* = 7.4 Hz, H-1′′ glucuronic acid), 3.38 (d, *J* = 9.2, H-5′′ glucuronic acid), ^13^C-NMR (DMSO-*d*_6_) δ (ppm): 157.61 (C-2), 134.05 (C-3), 177.57 (C-4), 160.92 (C-5), 98.95 (C-6), 164.92 (C-7), 93.78 (C-8), 156.50 (C-9), 103.55 (C-10), 120.61 (C-1′), 118.02 (C-2′), 144.79 (C-3′), 148.42 (C-4′), 115.40 (C-5′), 120.45 (C-6′), 103.08 (C-1′′), 73.95 (C-2′′), 76.63 (C-3′′), 71.78 (C-4′′), 74.16 (C-5′′), 172.20 (C-6′′).

Compound **6** (kaempferol-3-*O*-β-glucopyranoside = astragalin): ESI-MS negative: *m*/*z* 447 [M − H]^−^, *m*/*z* 285 [A − H]^−^, ESI-MS positive: *m*/*z* 449 [M + H]^+^, *m*/*z* 287 [A + H]^+^.^1^H-NMR (DMSO-*d*_6_) δ (ppm): 12.61 (s, 5-OH), 6.21 (d, *J* = 1.6 Hz, H-6), 6.43 (d, *J* = 1.6 Hz, H-8), 8.04 (d, *J* = 8.7 Hz, H-2′and H-6′), 6.88 (d, *J* = 8.7 Hz, H-3′and H-5′), 5.46 (d, *J* = 7,4 Hz, H-1′′ glucose); ^13^C-NMR (DMSO-*d*_6_) δ (ppm): 156.23 (C-2), 133.16 (C-3), 177.46 (C-4), 161.21 (C-5), 98.67 (C-6), 164.12 (C-7), 93.63 (C-8), 156.36 (C-9), 103.99 (C-10), 120.88 (C-1′), 130.88 (C-2′), 115,09 (C-3′), 159.94 (C-4′), 115.09 (C-5′), 130.88 (C-6′), 100.82 (C-1′′), 74.20 (C-2′′), 77.49 (C-3′′), 69.87 (C-4′′), 76.40 (C-5′′), 60.81 (C-6′′).

Compound **7** (quercetin-3-*O*-α-rhamnopyranosylo (1→6)-*β*-glucopyranoside = rutin): ESI-MS negative: *m*/*z* 609 [M − H]^−^, *m*/*z* 463 [M-rhamnosyl-H]^¯^, *m*/*z* 301 [A − H]^−^; ESI-MS positive: *m*/*z* 611 [M + H]^+^, *m*/*z* 465 [M-rhamnosyl + H]^+^, *m*/*z* 303 [A + H]^+^. ^1^H-NMR (DMSO-*d*_6_) δ (ppm): 12.59 (s, 5-OH), 6.18 (d, *J* = 2.1 Hz, H-6), 6.37 (d, *J* = 2.1 Hz, H-8), 7.52 (d, *J* = 2.1 Hz, H-2′), 6.83 (d, *J*= 8.2 Hz, H-5′), 7.54 (dd, *J* = 2.2 and *J* = 8.3 Hz, H-6′), 5.34 (d, *J* = 7.5 Hz, H-1′′ glucose), 4.38 (d, *J*= 1.2 Hz, H-1′′′ rhamnose), 0.99 (d, *J* = 6.2 Hz, H-6′′′ rhamnose), ^13^C-NMR (DMSO-*d*_6_) δ (ppm):156.42 (C-2), 133.27 (C-3), 177.32 (C-4), 161.20 (C-5), 98.70 (C-6), 164.23 (C-7), 93.59 (C-8), 156.56 (C-9), 103.88 (C-10), 121.13 (C-1′), 115.20 (C-2′), 144.75 (C-3′), 148.43 (C-4′), 116.22 (C-5′), 121.57 (C-6′), 101.18 (C-1′′), 74.05 (C-2′′), 75.88 (C-3′′), 69.98 (C-4′′), 76.42 (C-5′′), 66.97 (C-6′′), 100.73 (C-1′′′), 70.35 (C-2′′′), 70.53 (C-3′′′), 71.82 (C-4′′′), 68.23 (C-5′′′), 17.72 (C-6′′′).

#### 4.4.2. UPLC Analysis

##### Standard Solutions

The stock solutions (1 mg/mL) of phenolic acids: chlorogenic, caffeic, gallic, isovanilic, 4-hydroxybenzoic, and protocatechuic were freshly prepared in methanol. The working solutions were prepared by diluting the stock solution using mobile phase A. 2.0 μL volume at each concentration level of each phenolic acids working solutions (5–40 µg/mL) was injected eight times into the UPLC system for the construction of the calibration curves.

##### Sample Preparation

The GP extract was added to the SPE process. The cartridge (Oasis HLB Plus Short Cartridge, Waters, Milford, MA, USA) was conditioned with 5 mL of methanol followed by 5 mL of grade water. 1.0 mL of the GP extract was eluted with 50 mL of 70% methanol. The organic eluate was collected in a flask and then concentrated under the vacuum at 40°C to dryness, diluted with mixture methanol-water (1:1, *v*/*v*) to 2.0 mL, and then purified through a syringe filter (GHP, 25 mm, 0.2 µm, Waters).

##### Chromatographic Conditions

Identification and quantification of phenolic acids of GP extract were carried out by using an ultra-high performance liquid chromatography (UPLC, Acquity, Waters). The method was performed on an Acquity UPLC HSS T3 column (1.8 µm, 2.1 × 150 mm, Waters) with a guard pre-column of Acquity UPLC BEH C_18_ (1.7 µm, 2.1 × 5 mm, Waters) at the column temperature of 24 °C. Elution was conducted using mobile phase A: HPLC grade water and mobile phase B: HPLC grade acetonitrile. Each solvent (1000 mL) was acidulated by using formic acid (50 µL). The linear gradient was as follows: 3% to 13% B over 0.0 to 4.0 min, 13% to 17.5% B over 4.0 to 5.0 min, 17.5% B over 5.0 to 9.0 min, 17.5% to 3.0% B over 9.0 to 13.0 min with a flow rate of 0.275 mL/min. The spectral data of signals from the PDA detector were collected during the whole run in the range of 234 to 330 nm and processed by using Empower 2 Software Build 2154 (Waters, Milford, MA, USA). The presence of phenolic acids in the GP extract was confirmed by comparison of retention time and spectra of particular substances.

##### Validation of the UPLC Method

Quantitative analyses of phenolic acids by the UPLC-PDA were validated with respect to linearity, sensitivity, and precision. Linearity was evaluated by the correlation coefficient of the curves of studied compounds. A limit of detection (LOD) and quantification (LOQ) were determined by the injection of a series of dilutions of phenolic acids with LOD determined as the concentration that resulted in a peak area three times greater than the noise level and LOQ is a concentration that resulted in a peak area 10 times greater than the noise level. The precision of the method was estimated by running eight injections of six different concentrations, which were given on the same day, and the values of relative standard deviation were calculated to determine the intra-day precision.

#### 4.4.3. Total Phenolic Content (TPC)

TPC was determined using the Folin–Ciocalteu method [36] with minor modifications. A total of 0.2 mL of the extract (5 mg dry herb/mL) was mixed with 4.0 mL of water and 0.5 mL of Folin–Ciocalteu′s reagent. After 1 min, 2.0 mL of 20% sodium carbonate aq. solution was added and supplemented with distilled water to a total volume of 10 mL. The mixture was left for 30 min and then its absorbance was measured at 760 nm in pentaplicate. A blank sample of water and reagents was used as a reference. TPC was expressed as mg of chlorogenic acid equivalent per g of a dry herb utilizing a calibration curve of chlorogenic acid (y = 4.8882x; R^2^ = 0.9954) in a concentration range 0.002 mg/mL to 0.012 mg/mL.

#### 4.4.4. Total Flavonoid Content (TFC)

TFC was determined as described by Meda et al. [37]. Equal volumes of 2% AlCl_3_ in methanol the GP extract (3 mg/mL) were mixed and left for 10 min. The absorbance was measured in pentaplicate at 415 nm using a blank sample of water and methanol without AlCl_3_. TFC was expressed as mg of quercetin (6.25–100 mg/mL) per 100 g of dry weight (y = 0.0383x + 0.0599; R² = 0.9892).

### 4.5. Bioactivity Assay

#### 4.5.1. FRAP Assay

The FRAP assay was performed according to Tiveron el al. [38] with some modifications. The stock solutions of the FRAP reagent included 300 mM acetate buffer (pH 3.6), 10 mM TPTZ solution in 40 mM HCl, and 20 mM FeCl_3_·6H_2_O solution. The working FRAP solution was freshly prepared by mixing 25 mL acetate buffer, 2.5 mL TPTZ solution, and 2.5 mL FeCl_3_·6H_2_O solution and then warmed at 37 °C before usage. The examination of the GP extract (50 μL) was allowed to react with the FRAP solution (1500 μL) at 37 °C for 30 min. in the dark condition. Then the absorbance was read at 593 nm in pentaplicate. The standard curve was linear in the range of 10 to 100 μg Vitamin C/mL with R^2^ = 0.9998. The results were expressed as the IC_0.5_ (µg/mL), which corresponded to the extract concentration required to produce 0.5 O.D. value.

#### 4.5.2. Anti-Hyaluronidase Activity

Inhibition of hyaluronidase (HA) by the GP extract was determined by a turbidimetric method described by Grabowska et al. [39] with minor modifications. Additionally, 25 µL of enzyme (30 U/mL of acetate buffer pH 7.0), 25 µL of acetate buffer (50 mM, pH 7.0, with 77 mM NaCl and 1 mg/mL of albumin), 15 µL of acetate buffer (pH 4.5), and 10 µL solutions of the examined substances (2.3–25 mg/mL) were combined in order to produce reagent mixtures with the final concentrations of 0.23 to 2.50 mg/mL. All the reaction mixtures were incubated at 37 °C for 10 min. Next, 25 µL of HA (0.3 mg/mL of acetate buffer pH 4.5) was added and incubated at 37 °C for 45 min. The undigested HA was precipitated with the addition of 200 µL 2.5% CTAB in 2% NaOH (pH 12). The mixture was kept at room temperature for 10 min. Turbidance of the reaction mixture was measured as the absorbance at 600 nm (Multiskan GO 1510, Thermo Fisher Scientific, Vantaa, Finland). Kaempferol was used as the positive control (final concentration 0.5–1.0 mg/mL). The absorbance in the absence of the enzyme was used as the blind control. To exclude nonspecific changes in turbidity (caused by other factors rather than not digested HA), the tests without HA solution were conducted. For the investigated extract, three independent experiments were carried out in triplicate. The percentage of inhibition was calculated by using the equation below.

(1) $$\%\;\mathrm{inhibition}\;\mathrm{activity}=\frac{T_{S}-T_{C}}{T_{H}-T_{c}}\cdot 100\%$$

*T_S_*—absorbance of the enzyme + substrate (HA) + substance sample. *T_C_*—absorbance of the enzyme + substrate (HA). *T_H_*—absorbance of the HA + substance sample.

#### 4.5.3. Cell Culture

The experiments were performed using human umbilical vein endothelial cells (HUVECs) of the EA.hy926 line (kindly donated by Dr. Edgell, University of North Carolina, Chapel Hill, NC, USA) [40]. Cells were routinely maintained in the Earl′s-buffered M199 culture medium, supplemented with amphotericin (2.5 μg/mL), gentamycin (50 μg/mL), l-glutamine (2 mM), hydrocortisone (0.4 μg/mL), and 10% *v*/*v* fetal calf serum (ThermoFisher Scientific, Waltham, Milford, MA, USA).

To ensure that the GP extract produced was not contaminated with endotoxin, GP extract was tested with the Limulus amebocyte lysate (LAL) assay.

#### 4.5.4. Cell Viability

The viability of cells treated with GP (0.125–1 mg/mL) was determined using the Trypan blue exclusion test. Cells were suspended in Hanks′ Balanced Salt Solution and mixed 1:1 (*v*/*v*) with 0.4% Trypan blue solution. After 10 min, the number of blue-stained non-viable cells was counted in a hemocytometer.

#### 4.5.5. Cell Proliferation

Cell proliferation was measured using the MTT assay [41]. Following exposure to the GP extract, cells were incubated for 4 h at 37 °C with 1.25 mg/mL MTT salt (3-[4.5-dimethylthiazol-2-yl]-2.5-diphenyl-tetrazolium bromide). The formazan product generated was dissolved with acidic solution of 20% *w*/*v* sodium dodecyl sulphate and 50% *v*/*v* *N*,*N*-dimethylformamide. The absorbance of the converted dye was recorded at 595 nm with a reference wavelength of 690 nm.

#### 4.5.6. Cytokine Measurements

Endothelial cells were exposed to GP extract (0.5–1 mg/mL) in the presence or absence of IL-1β (1 ng/mL). After 24 h, the medium was collected and frozen at −80°C until it was assayed. The cells were lysed with 0.1 N NaOH and measured for protein concentration with the Bradford method using the Protein Assay Dye Reagent. IL-6 concentrations were measured with the DuoSet Immunoassay Development Kits (R&D Systems, Minneapolis, MN, USA) as per manufacturer′s instructions. The sensitivity of the assay was estimated to be 2.6 pg/mL.

#### 4.5.7. Wound Healing

The assay was performed as described previously [42,43]. The cells were grown to confluence and then scratched with a cell scraper (Nunc). The resulting debris was removed by gentle washing with medium. After that, cells were maintained for up to 12 h in standard culture medium with either GP (0.5 to 1.0 mg/mL) or vehicle control in an incubator coupled to an Axio Observer D1 inverted microscope (Zeiss). The images of the closing wound were acquired by time-lapse microscopy at 30-min intervals and analyzed using the AxioVision Rel. 4.6.3 image analysis software (Zeiss). The data were expressed as the percentage of regeneration in each time interval.

The wound scratch assay is a well-established method to study wound healing in vitro and is thought to be particularly suited for measuring cell migration [44].

#### 4.5.8. Measurement of Reactive Oxygen Species (ROS)

The generation of ROS by endothelial cells treated with the GP extract (0.5 to 1.0 mg/mL) was assessed by labeling with 2′–7′-dichlorodihydrofluorescein diacetate (H_2_DCFDA) that is trapped inside the cells and activated by intracellular ROS. Following the exposure to test solutions, 2 × 10^4^ cells were loaded with 10 µM H_2_DCFDA for 30 min and then treated with the lysis buffer. Fluorescence emitted by cell lysates was measured in a Wallac Victor spectrofluorometer (Perkin-Elmer, Finland) using wavelengths of 485 nm and 535 nm for excitation and emission, respectively. During the experiments of ROS detection in vitro, the internal positive control (100 µM H_2_O_2_) was also detected. In addition, the HUVEC line and EA.hy926 line were incubated ± GP extract (0.5–1.0 mg/mL) for 24 h in 6 well dishes. Trypsinized cells (2.5–3 × 10^5^) were washed twice in cold PBS and dihydrorhodamine 123 (10 μg/mL) was added. After 30 min of incubation at 37 °C, the samples were measured by a BDFACS Aria III cytometer and results were analyzed by FACS Diva Software. Dihydrorhodamine 123 (DHR) infiltrates the cell membrane and, after oxidation by ROS, is converted to rhodamine 123, which emits a green fluorescent signal after excitation by a blue laser (λ = 488 nm). ROS levels were measured as the difference in median fluorescence intensity (MdFI). As a control, we used cells with correct concentrations of GP extract without DHR.

#### 4.5.9. Statistical Analysis

Statistical analysis was performed with an analysis of variance and post-hoc testing using GraphPad Prism™ 6.00 software (Graph Pad Software Inc., San Diego, CA, USA). Results were expressed as means ± SD. A *p* value of less than 0.05 was considered significant. The IC_50_ values were calculated with Prism™ 6.0 software using nonlinear regression.

## 5. Conclusions

The anti-inflammatory activity of the rich polyphenol extract from GP herb is, at least in part, related to free radical scavenging activity, inhibition of IL-6 release, and an inhibitory effect on hyaluronidase activity. The results of this study demonstrate that such a plant extract could have a potential as a drug in the treatment of skin disorders.

## Figures and Tables

**Table 1:** Regression data, LODs, LOQs, and recovery for studied compounds.

Phenolic Acid	Regression Equation	R^2^	Linear Range (μg/mL)	LOD (μg/mL)	LOQ (μg/mL)	Recovery (%)
gallic	y = 88,398x − 53,935	0.9999	5–40	0.45	1.34	71.74 ± 0.02
protocatechuic	y = 104,765x − 45,841	0.9999	5–40	0.25	0.75	90.03 ± 0.05
chlorogenic	y = 79,806x − 60,132	0.9998	5–40	0.74	2.22	83.63 ± 0.01
4-hydroxybenzoic	y = 184,507x − 6297	0.9994	5–40	0.75	2.24	90.61 ± 0.07
caffeic	y = 145,745x − 104,383	0.9995	5–40	0.63	1.89	84.42 ± 0.05
isovanilic	y = 112,849x − 67,266	0.9999	5–40	0.25	0.76	90.39 ± 0.06

## Supplementary Materials

Supplementary materials are available online.
