# Supplementary material for: Anti-inflammatory Activity and Phytochemical Profile of Galinsoga Parviflora Cav
Source: Molecules. 2018 Aug 24;23(9):2133. doi: 10.3390/molecules23092133 (PMC6225198; doi:10.3390/molecules23092133)
Supplement: Supplementary file 1 [file molecules-23-02133-s001.pdf]

# Supplementary Materials

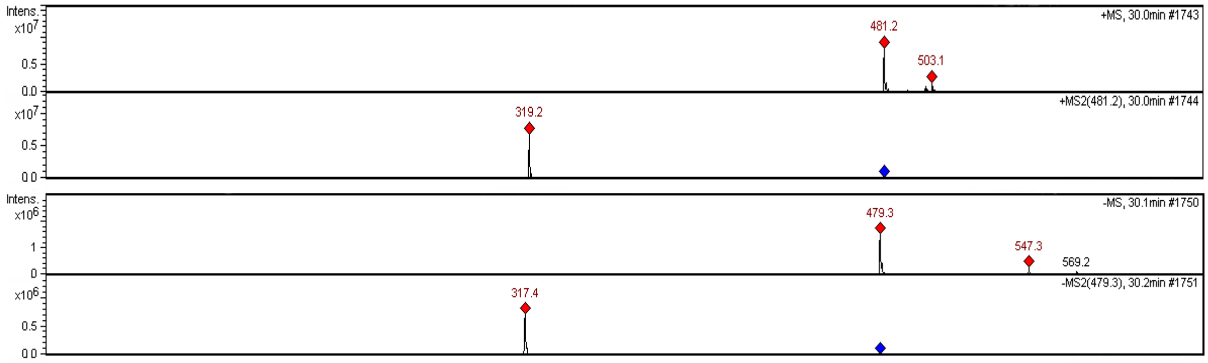


**Figure S1.** Mass spectrum of **compound 1**.

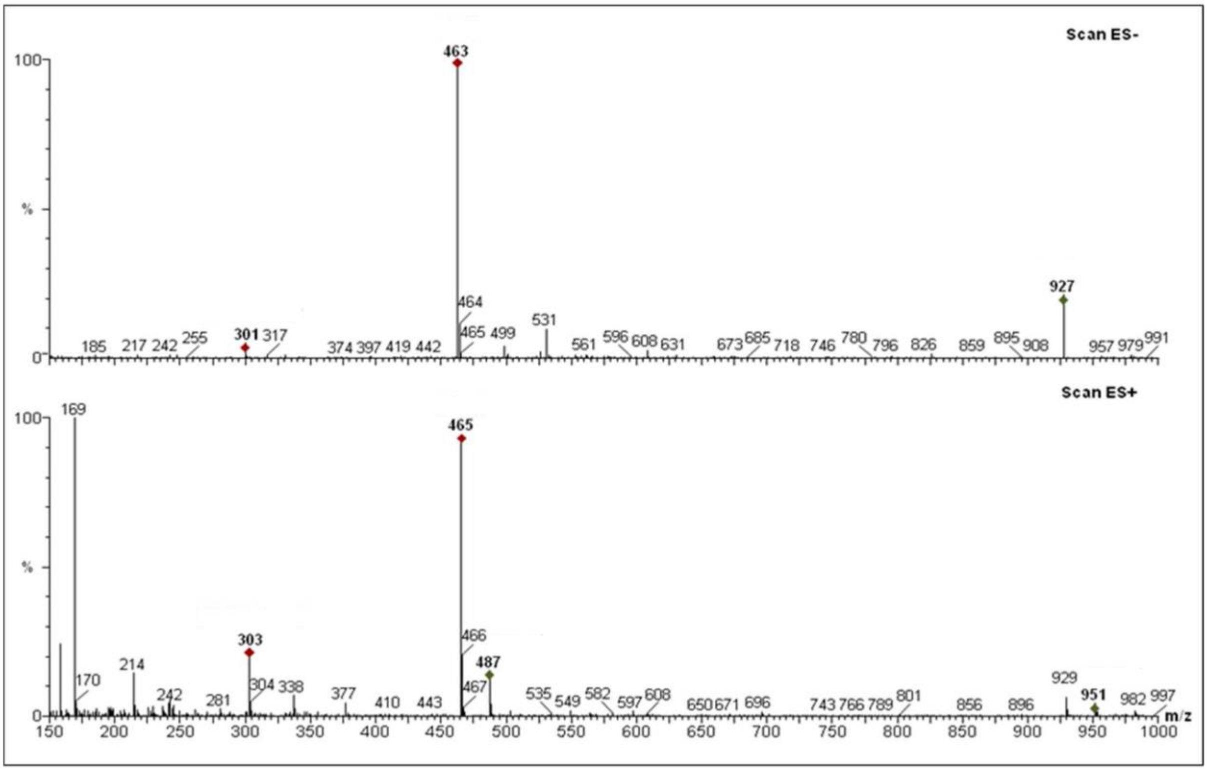


**Figure S2.** Mass spectrum of **compound 2**.

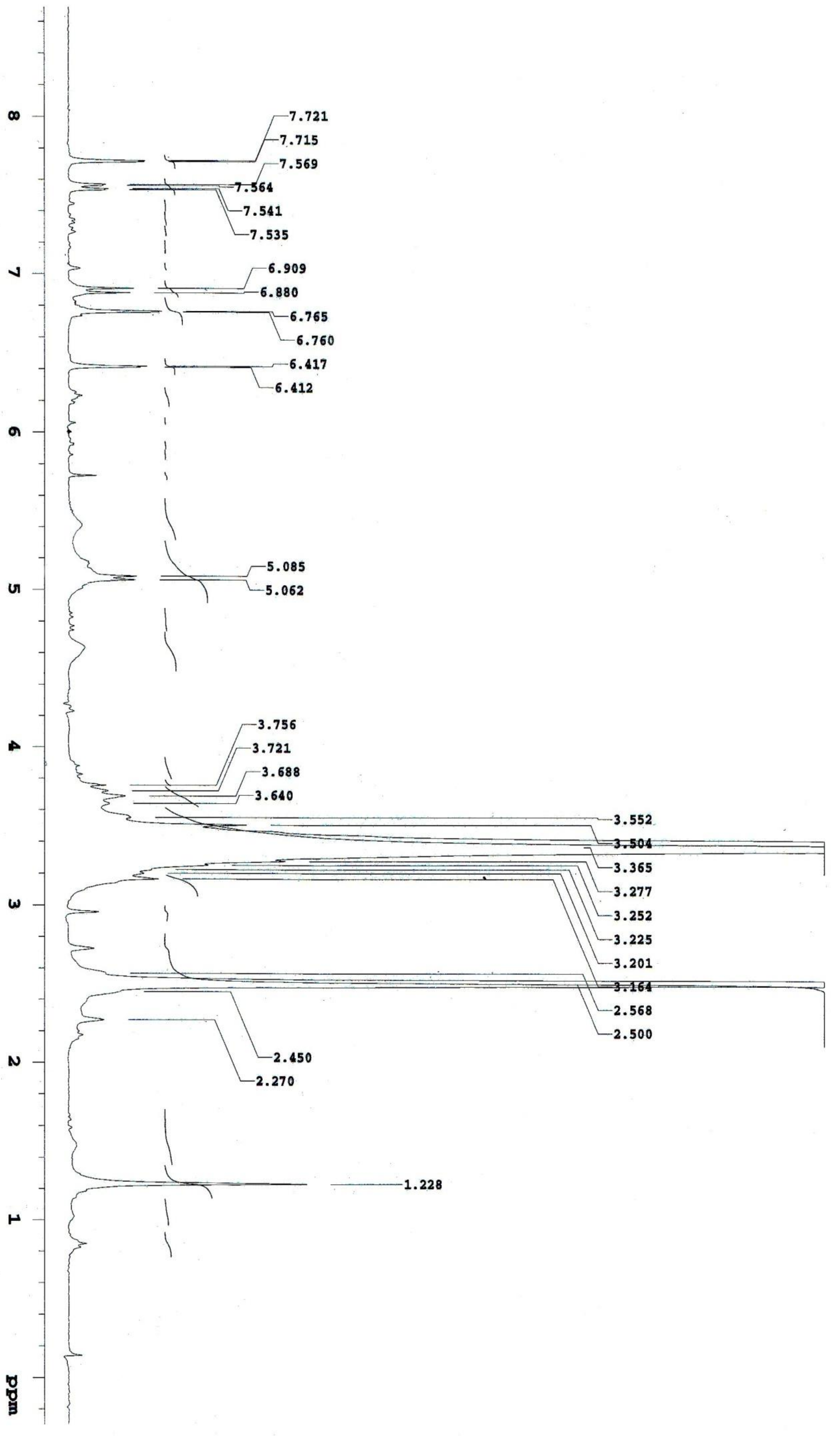


**Figure S3.** $^1$H NMR spectrum of **compound 2**.

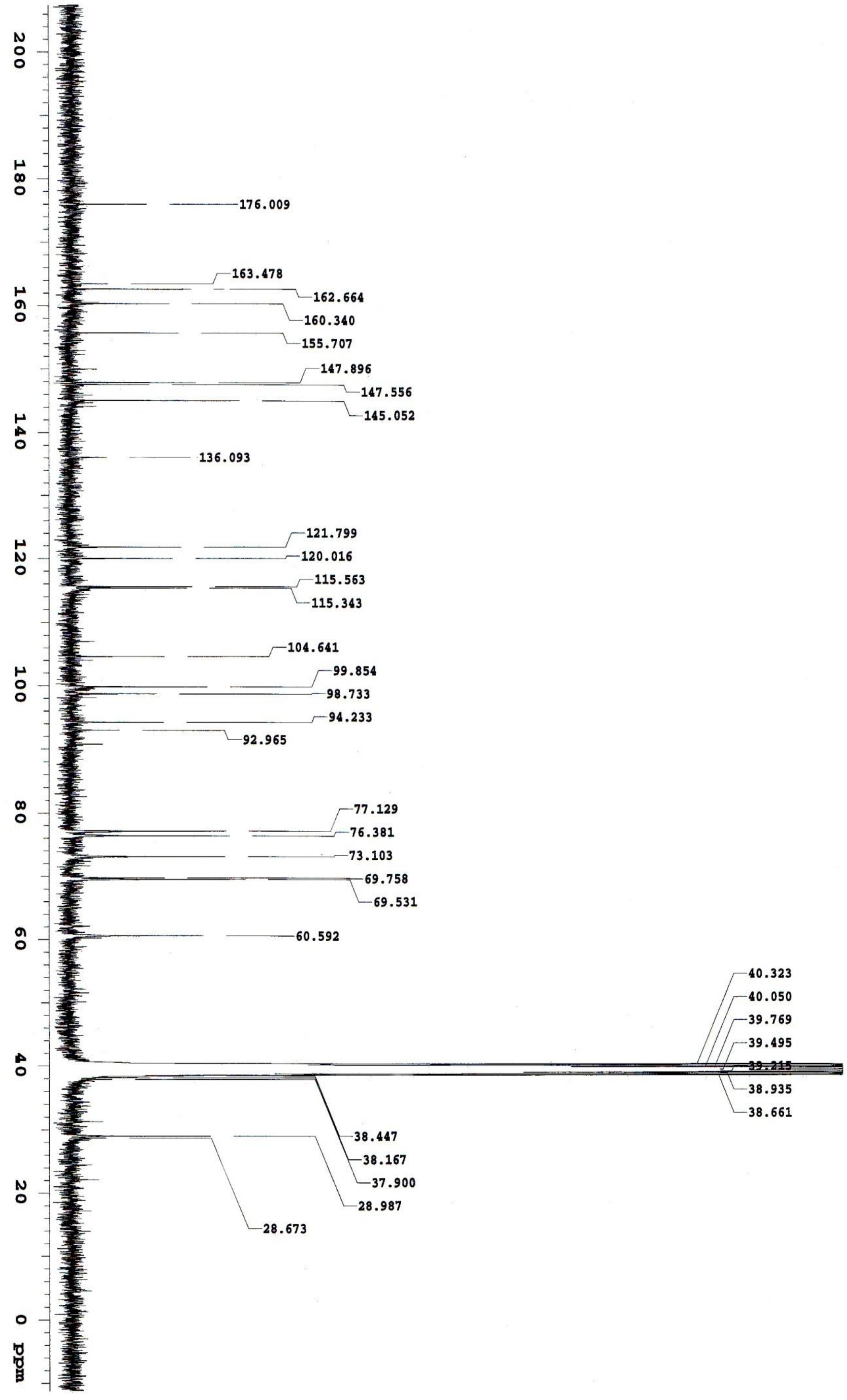


**Figure S4.** $^{13}C$ NMR spectrum of **compound 2**.

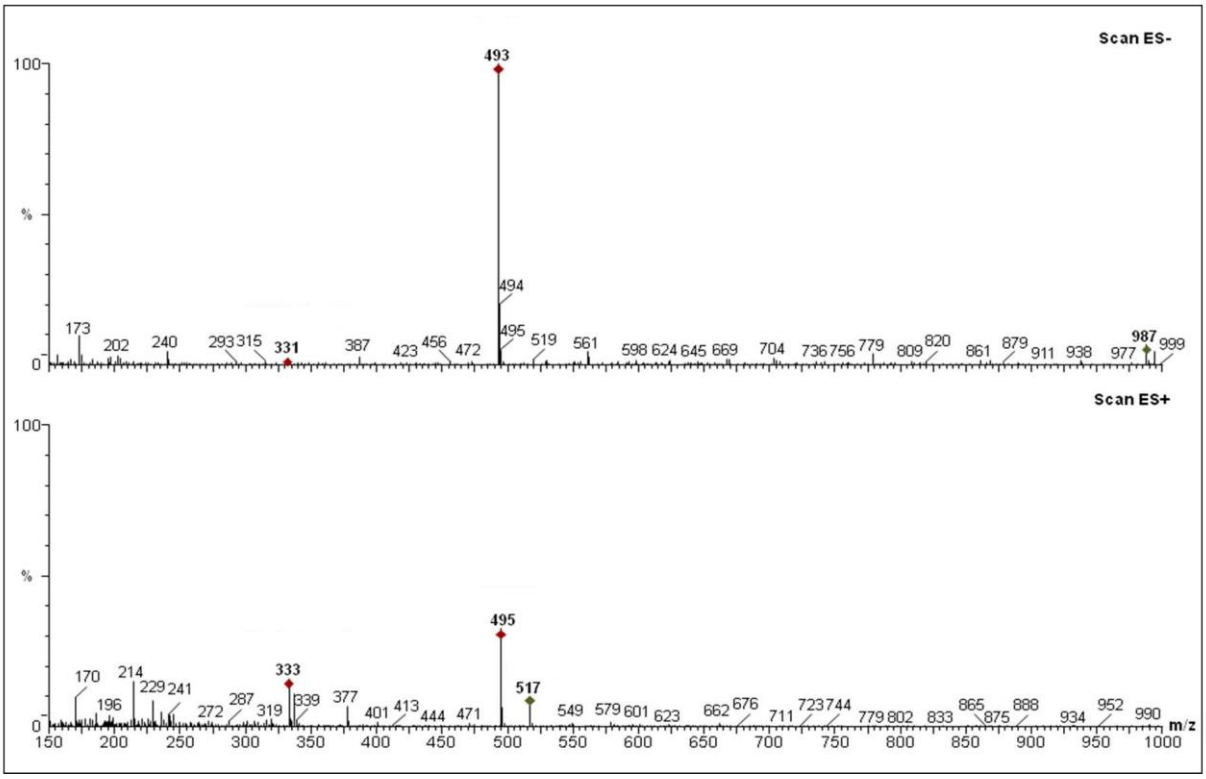


**Figure S5.** Mass spectrum of **compound 3**.

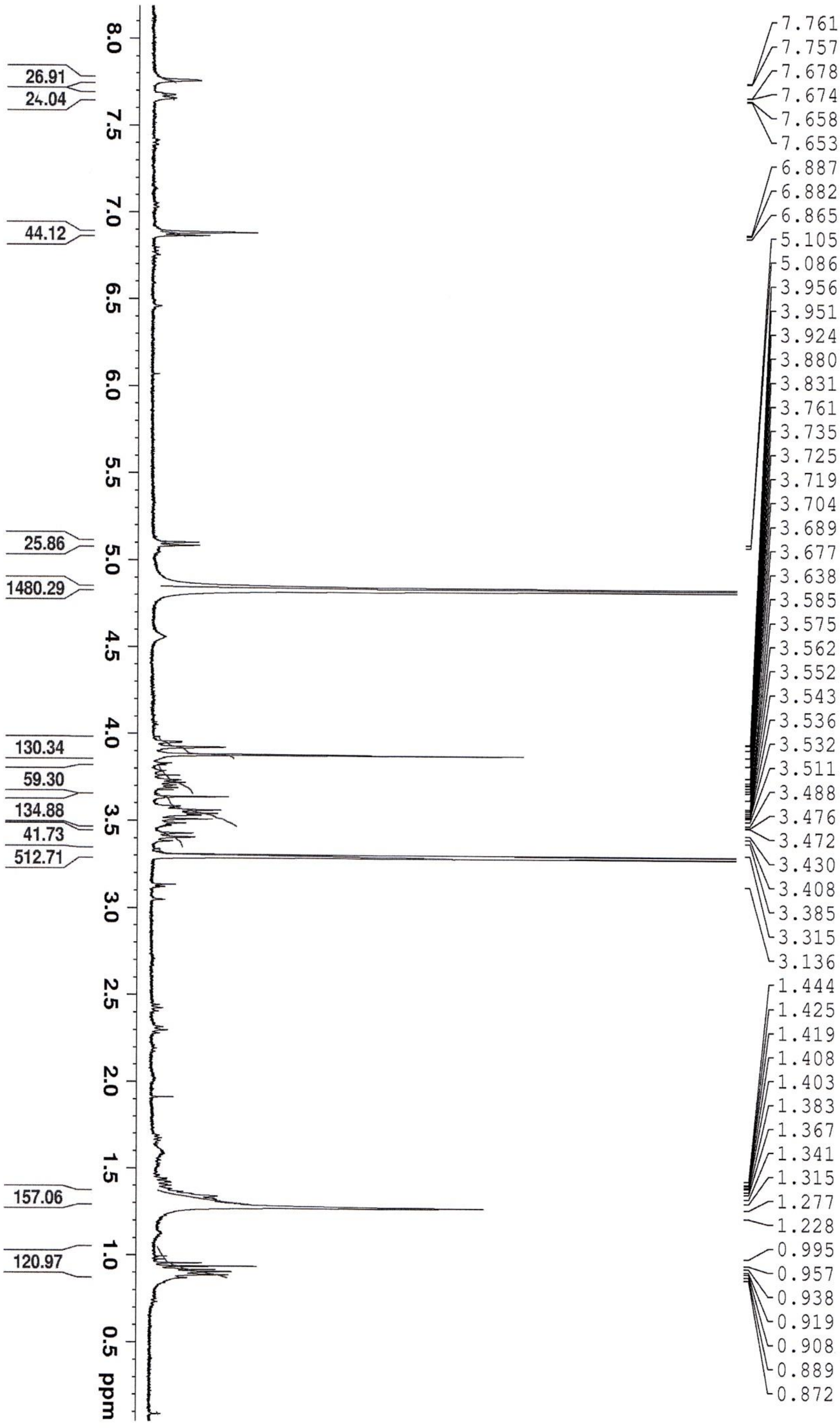


**Figure S6.** $^{1}H$ NMR spectrum of **compound 3**.

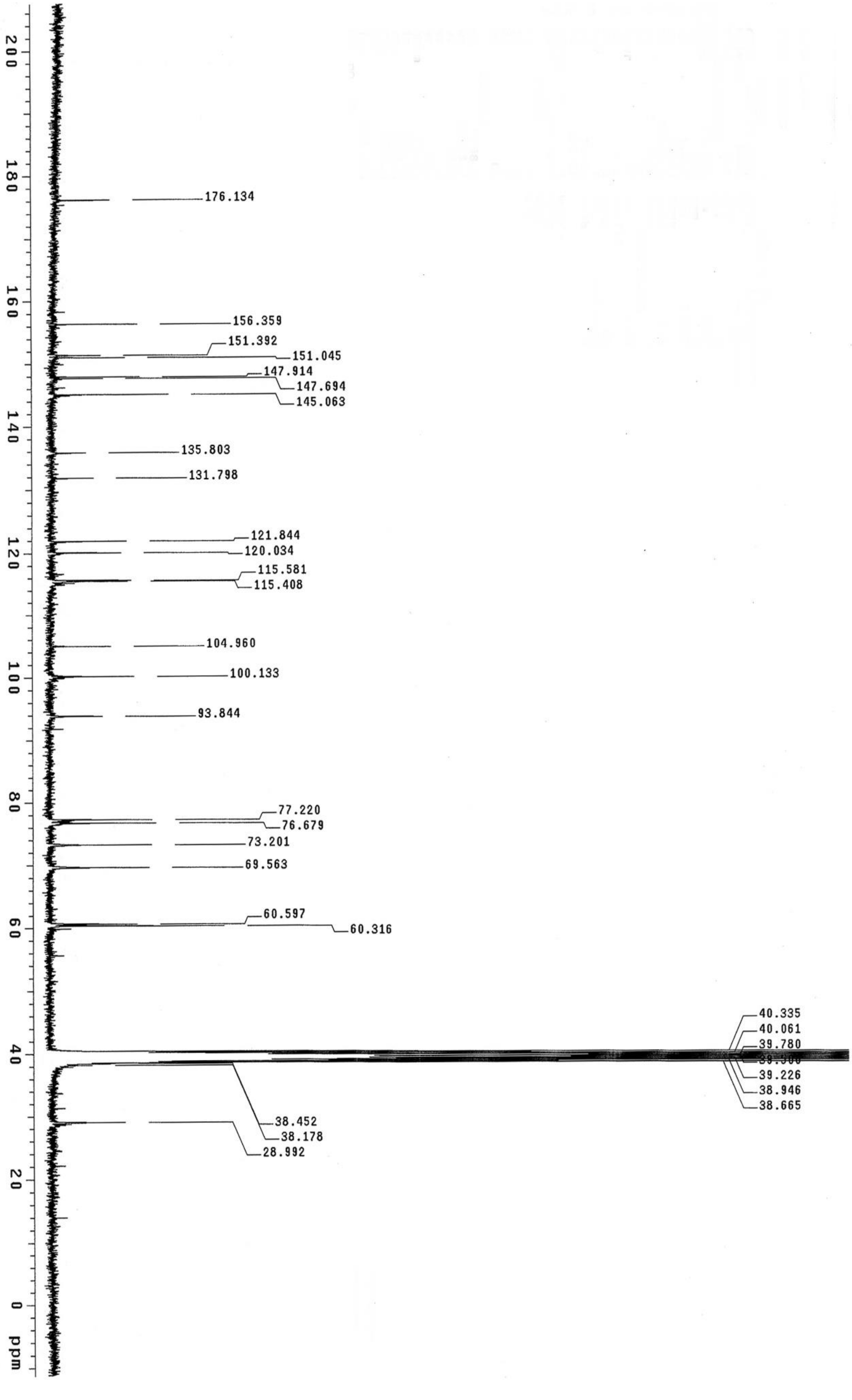


**Figure S7**. $^{13}$C NMR spectrum of **compound 3**.

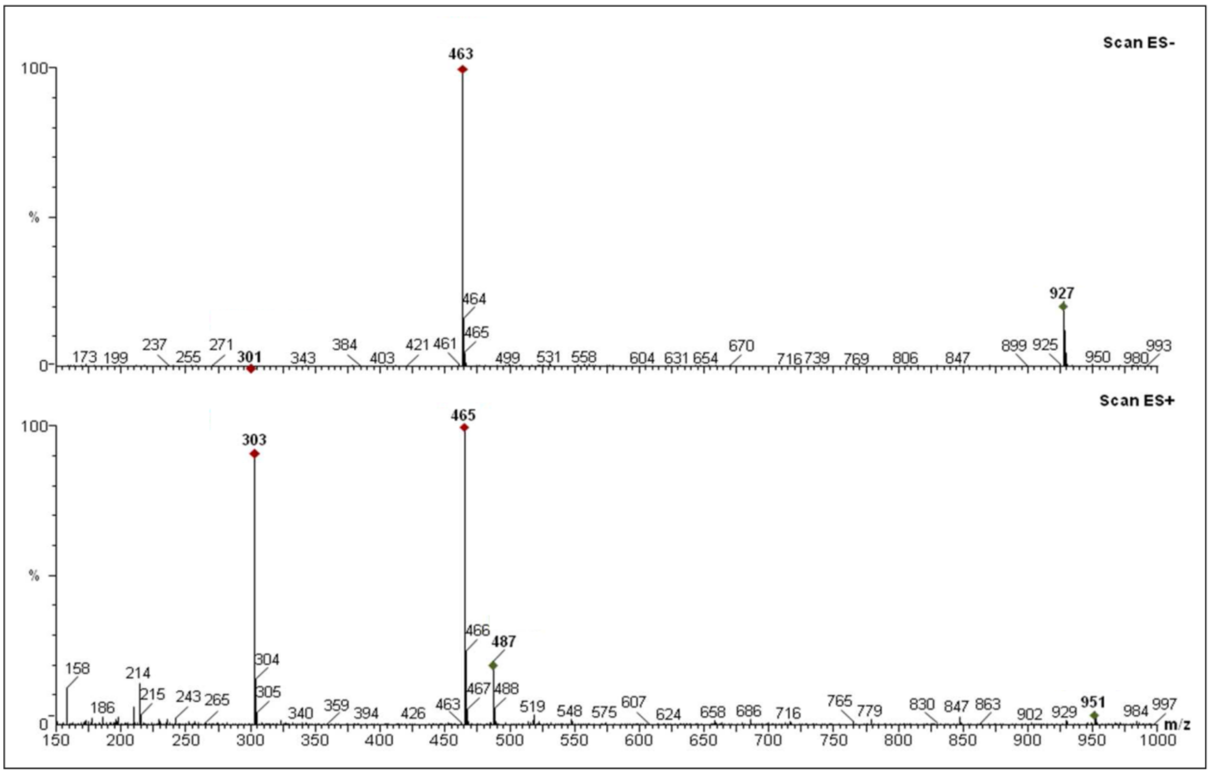


**Figure S8.** Mass spectrum of **compound 4.**

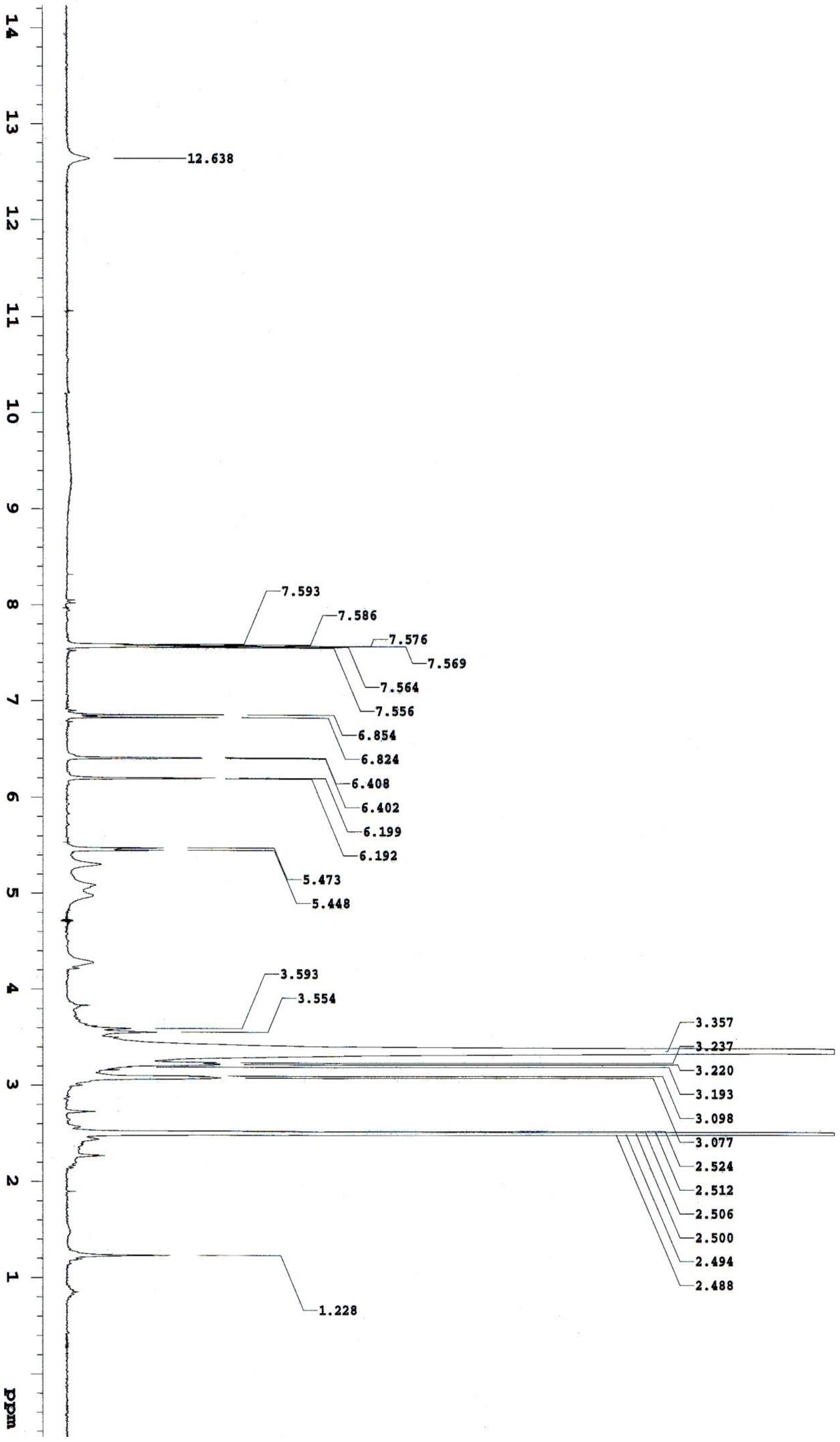


**Figure S9.** $^1H$ NMR spectrum of **compound 4**.

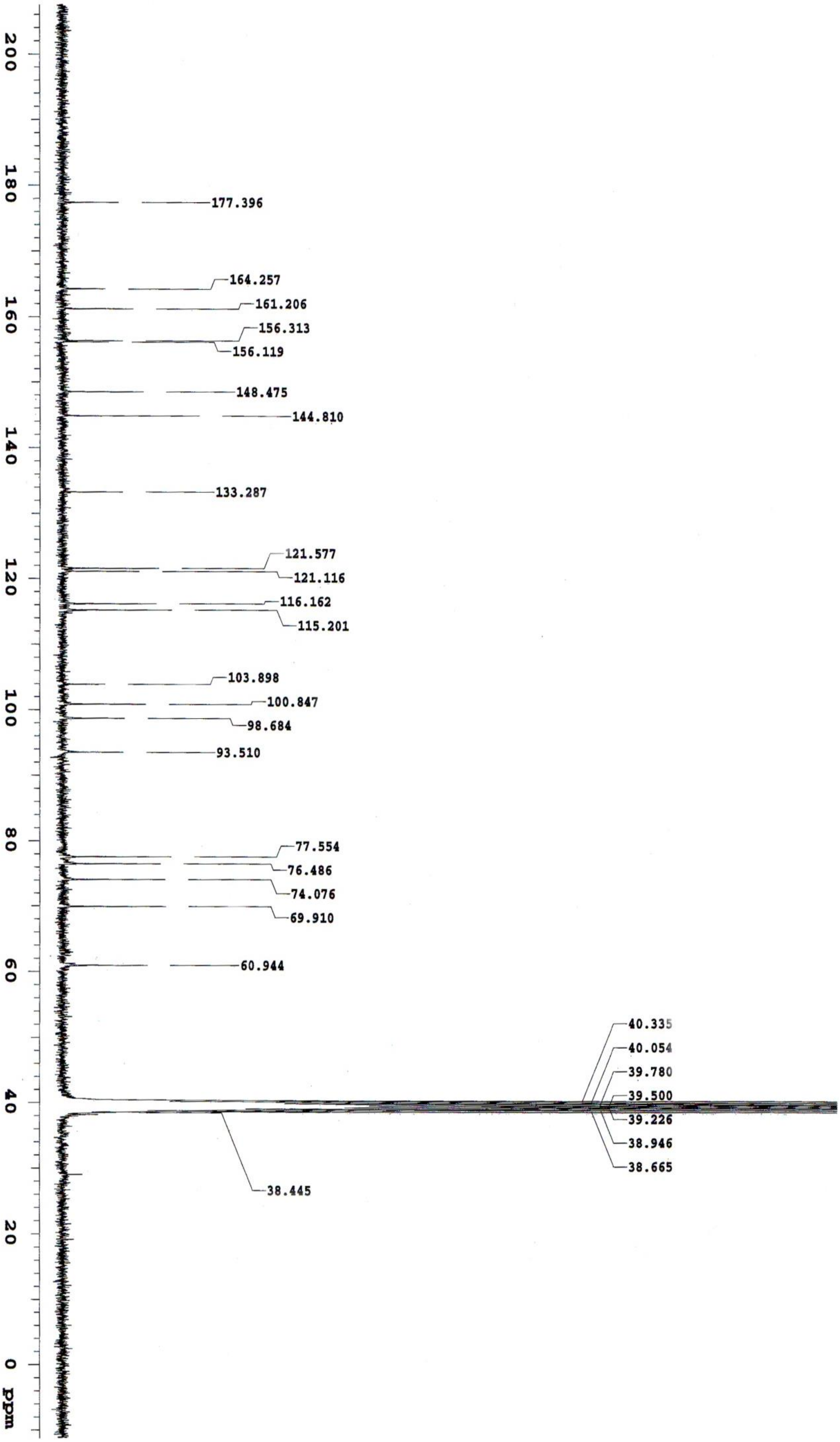


**Figure S10.** $^{13}C$ NMR spectrum of **compound 4**.

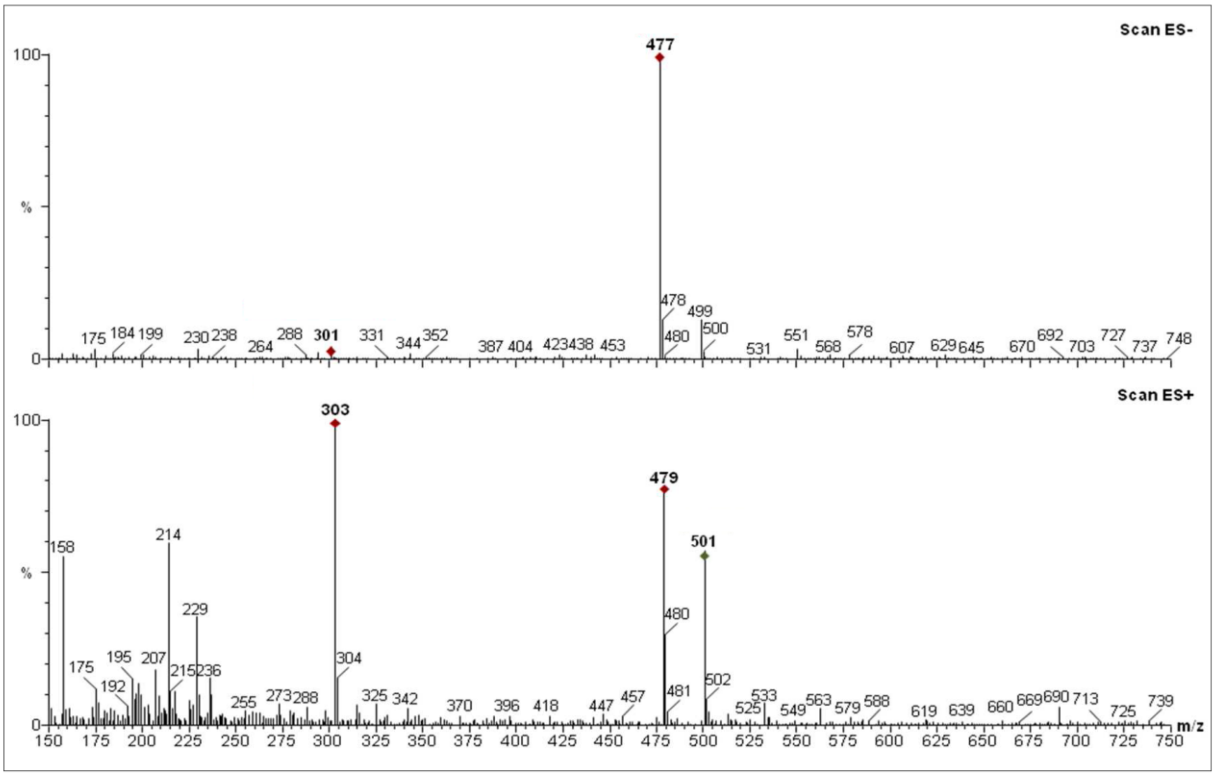


**Figure S11.** Mass spectrum of **compound 5**.

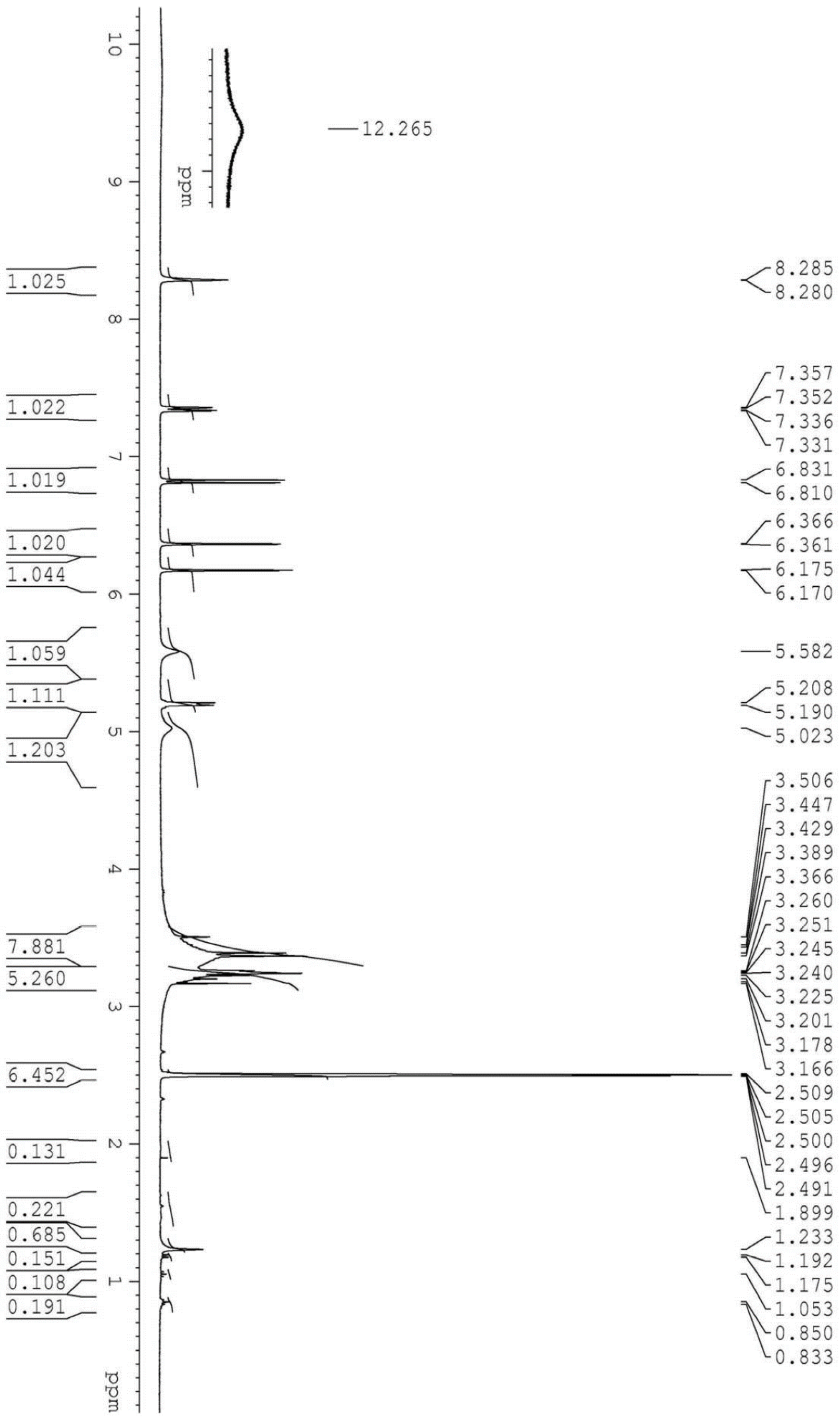


**Figure S12.** $^{1}$H NMR spectrum of **compound 5**.

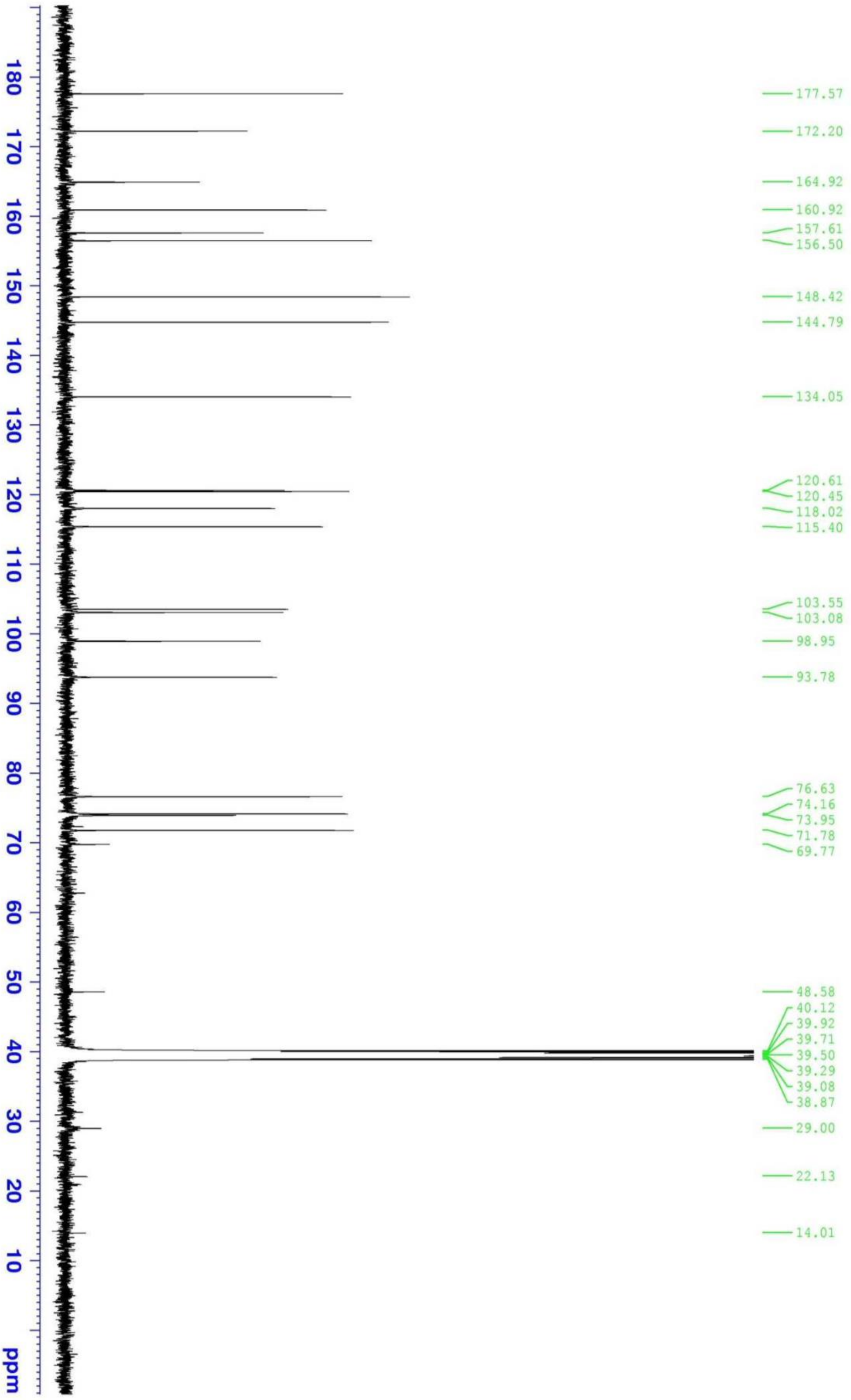


**Figure S13.** $^{13}$C NMR spectrum of **compound 5**.

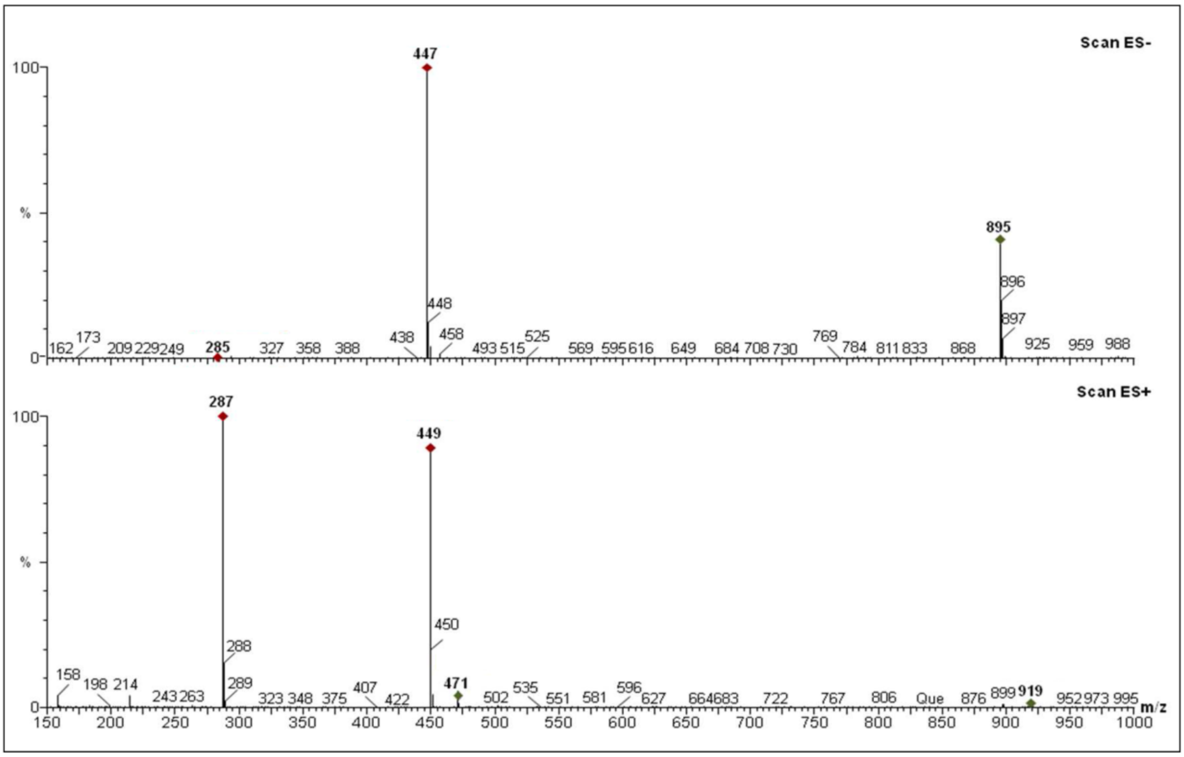


**Figure S14.** Mass spectrum of **compound 6**.

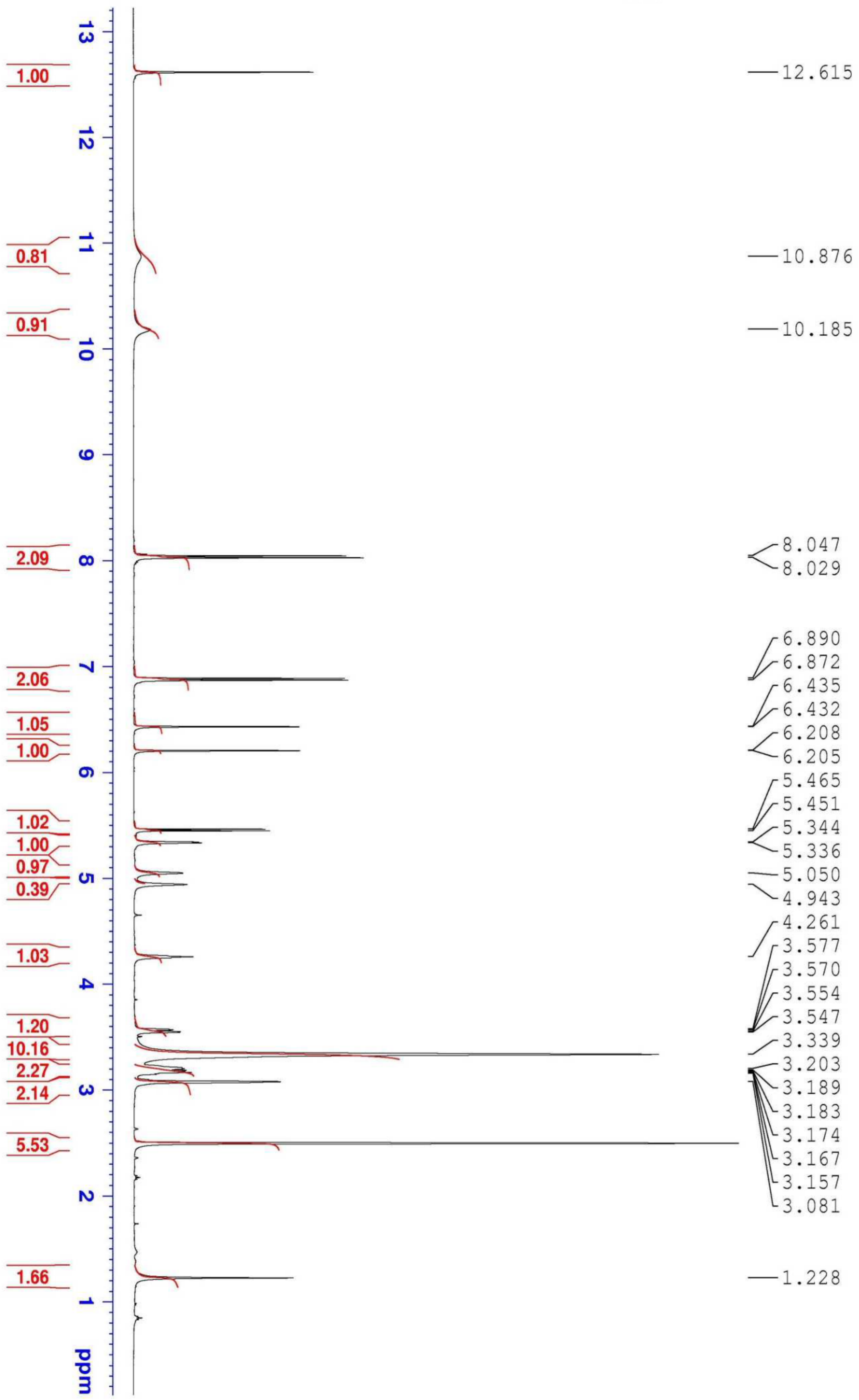


**Figure S15.** $^{1}$H NMR spectrum of **compound 6**.

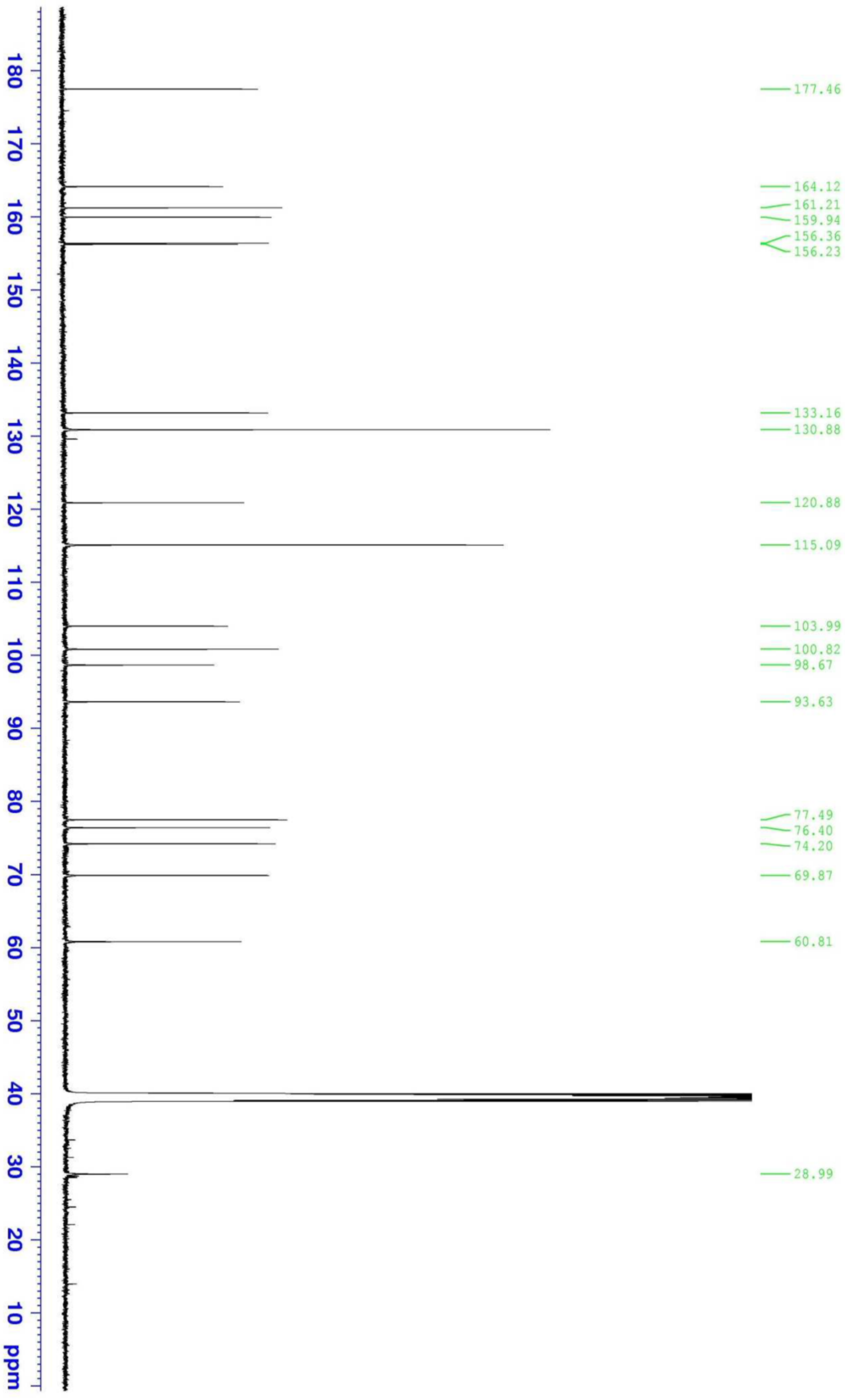


**Figure S16.** $^{13}C$ NMR spectrum of **compound 6**.

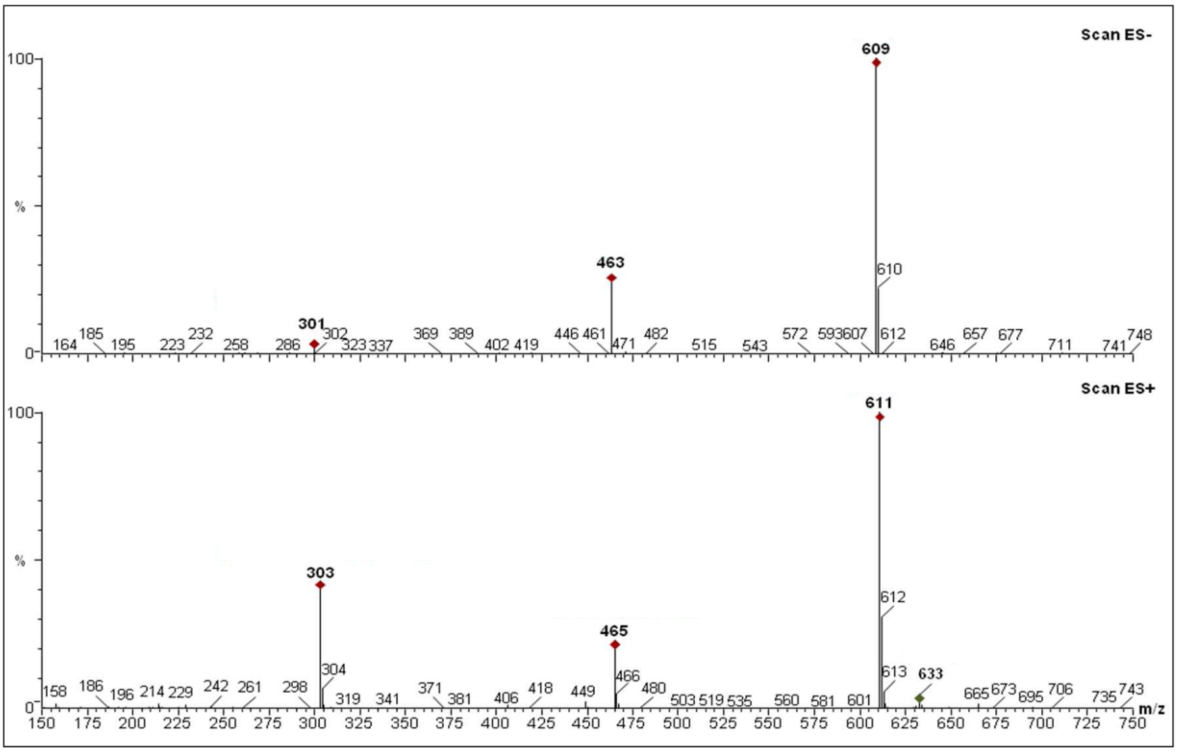


**Figure S17.** Mass spectrum of **compound 7.**

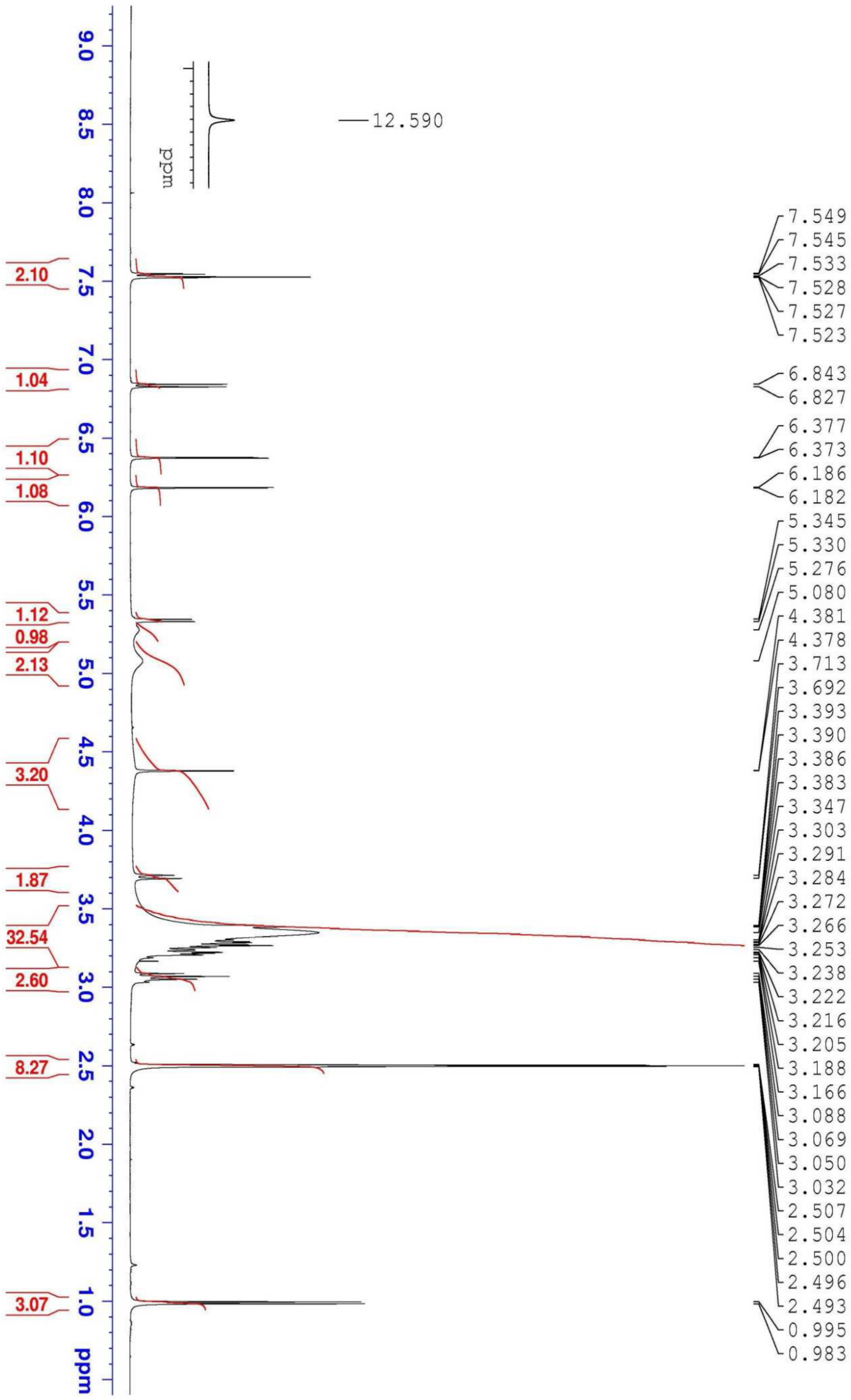


**Figure S18.** $^{1}$H NMR spectrum of **compound 7**.

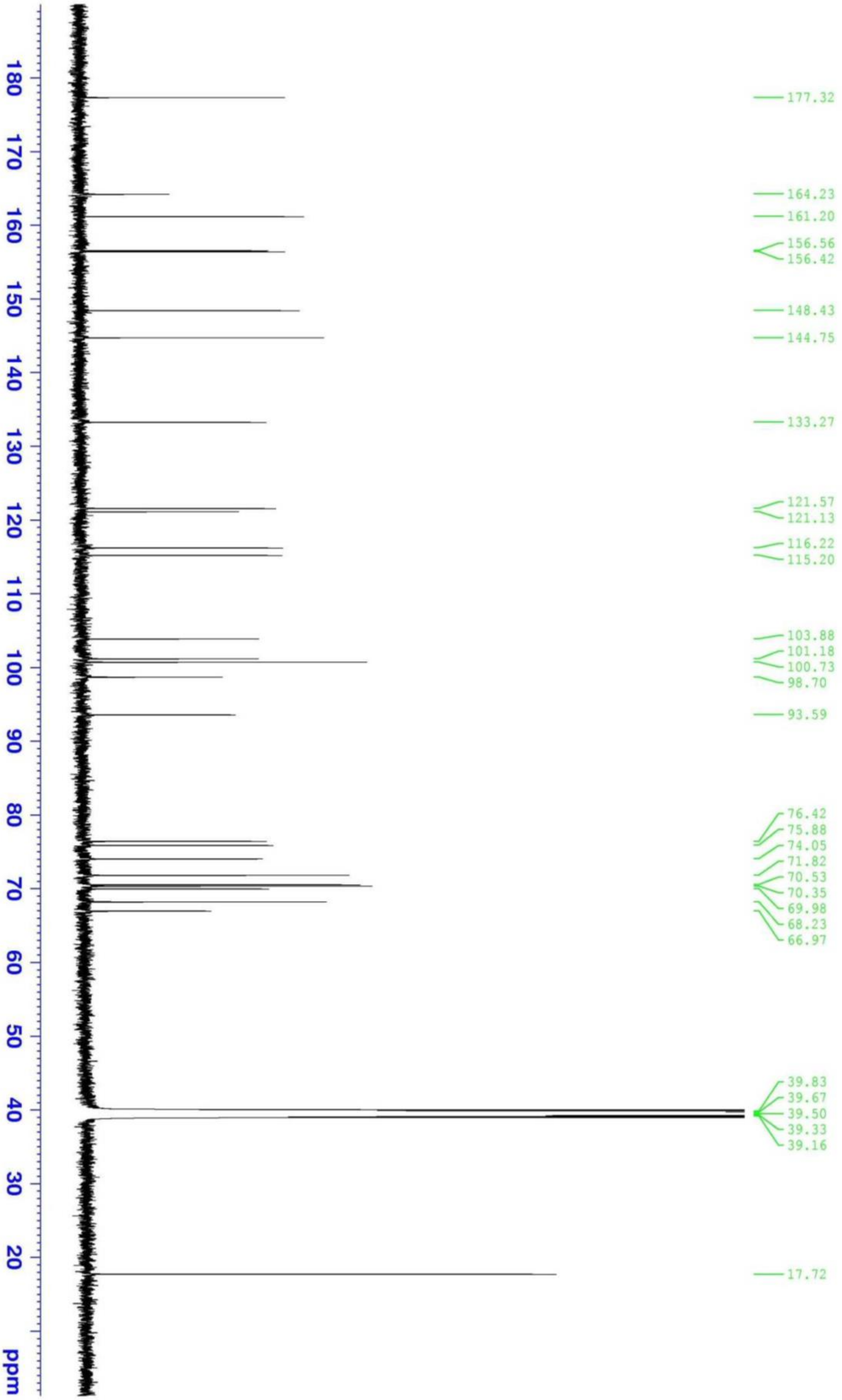


**Figure S19.** $^{13}C$ NMR spectrum of **compound 7**.
